# SIRT6-regulated macrophage efferocytosis epigenetically controls inflammation resolution of diabetic periodontitis

**DOI:** 10.7150/thno.78878

**Published:** 2023-01-01

**Authors:** Bang Li, Zhili Xin, Shiyu Gao, Yangjie Li, Songsong Guo, Yu Fu, Rongyao Xu, Dongmiao Wang, Jie Cheng, Laikui Liu, Ping Zhang, Hongbing Jiang

**Affiliations:** 1 Jiangsu Key Laboratory of Oral Diseases, Nanjing Medical University, Nanjing 210029, Jiangsu Province, China.; 2 Department of Oral and Maxillofacial Surgery, Affiliated Hospital of Stomatology, Nanjing Medical University, Nanjing 210029, Jiangsu Province, China.; 3 Department of Basic Science of Stomatology, Affiliated Hospital of Stomatology, Nanjing Medical University, Nanjing 210029, Jiangsu Province, China.; 4 Jiangsu Province Engineering Research Center of Stomatological Translational Medicine, Nanjing 210029, Jiangsu Province, China.

**Keywords:** SIRT6, Macrophage efferocytosis, Neutrophil extracellular trap, Inflammation resolution, Diabetes, Periodontitis

## Abstract

**Rationale:** Diabetes exacerbates the prevalence and severity of periodontitis, leading to severe periodontal destruction and ultimately tooth loss. Delayed resolution of inflammation is a major contributor to diabetic periodontitis (DP) pathogenesis, but the underlying mechanisms of this imbalanced immune homeostasis remain unclear.

**Methods:** We collected periodontium from periodontitis with or without diabetes to confirm the dysfunctional neutrophils and macrophages in aggravated inflammatory damage and impaired inflammation resolution. Our *in vitro* experiments confirmed that SIRT6 inhibited macrophage efferocytosis by restraining miR-216a-5p-216b-5p-217 cluster maturation through ''non-canonical'' microprocessor complex (RNA pulldown, RIP, immunostaining, CHIP, Luciferase assays, and FISH). Moreover, we constructed m6SKO mice that underwent LIP-induced periodontitis to explore the *in vitro* and *in vivo* effect of SIRT6 on macrophage efferocytosis. Finally, antagomiR-217, a miRNA antagonism, was delivered into the periodontium to treat LIP-induced diabetic periodontitis.

**Results:** We discovered that insufficient SIRT6 as a histone deacetylase in macrophages led to unresolved inflammation and aggravated periodontitis in both human and mouse DP with accumulated apoptotic neutrophil (AN) and higher generation of neutrophil extracellular traps. Mechanistically, we validated that macrophage underwent high glucose stimulation resulting in disturbance of the SIRT6-miR-216/217 axis that triggered impeded efferocytosis of AN through targeting the DEL-1/CD36 axis directly. Furthermore, we demonstrated the inhibitory role of SIRT6 for MIR217HG transcription and identified a non-canonical action of microprocessor that SIRT6 epigenetically hindered the splicing of the primary miR-216/217 via the complex of hnRNPA2B1, DGCR8, and Drosha. Notably, by constructing myeloid-specific deletion of SIRT6 mice and locally delivering antagomir-217 in DP models, we strengthened the* in vivo* effect of this axis in regulating macrophage efferocytosis and inflammation resolution in DP.

**Conclusions:** Our findings delineated the emerging role of SIRT6 in mediating metabolic dysfunction-associated inflammation, and therapeutically targeting this regulatory axis might be a promising strategy for treating diabetes-associated inflammatory diseases.

## Introduction

It is well-recognized that diabetes mellitus is an age-related disease characterized by insulin resistance, hyperglycemia, and inflammatory disorder, which leads to an increased risk and severity of complications due to prolonged inflammatory responses 1, 2. Diabetes, especially type 2 diabetes mellitus (T2DM, accounting for 90% of the cases), obviously enables severe periodontitis with the characteristics of high morbidity, unresolved inflammation, and ultimately tooth loss 3, 4. Compared with chronic periodontitis (CP), diabetic periodontitis (DP) exhibits more severe destruction of periodontium 5. Meanwhile, unlike dental plaque directly acting on CP, the high glucose environment in DP leads to the dysregulation of the host immune-inflammatory response, consequently provoking persistent inflammation and accelerating periodontal destruction 2, 6. In addition, DP might adversely promote systemic inflammation by influencing glycemic control and increasing the risk of other diabetes complications, such as atherosclerosis and Alzheimer's disease 3, 6, 7. Thus, periodontitis and diabetes, two of the most common inflammatory diseases, are linked to bidirectional risk via local and systemic inflammatory responses.

In healthy, most inflammatory responses are self-limiting with the endogenous host successfully orchestrating the onset and resolution of inflammation to maintain biological homeostasis. As an essential part of the innate immune system, circulating neutrophils are first recruited in the infectious or trauma region to clear the microorganisms or dead cells by executing the function of phagocytosis or releasing neutrophil extracellular traps (NETs) 4, 8, 9. Subsequently, neutrophils undergo spontaneous apoptosis and immediate efferocytosis by infiltrating macrophages to prevent secondary necrotic cells from releasing damage-associated molecular patterns (DAMP) 10, 11. However, chronic non-healing or slow-healing wounds in diabetic individuals are often associated with prolonged inflammation and neutrophil persistence 12. In addition, with the biological behavior of efferocytosis, numerous anti-inflammatory molecules like TGF-β, IL-10, and PGE2 are secreted from macrophages, suggesting that this process is necessary for promoting inflammation resolution and tissue repair 13-15. Notably, Kourtzelis et al. report that developmental endothelial locus-1 (DEL-1) functions as a downstream effector of macrophage efferocytosis in the inflammation resolution of periodontitis 16. Recently, emerging evidence highlights the roles of impaired efferocytosis in a variety of inflammatory diseases including diabetic foot ulcers, pulmonary fibrosis, cystic fibrosis, and atherosclerosis 10-14, 17, while the physiological functions of efferocytosis in DP have not been completely established.

SIRT6 as an NAD^+^-dependent histone deacetylase has recently attracted much attention as it confers the transcriptionally regulatory function in glucose metabolism, inflammatory homeostasis, and longevity via removing acetylation from histone 3 lysine 9 (H3K9) and histone 3 lysine 56 (H3K56) 18-20. Noticeably, myeloid cell-specific SIRT6 deficiency delays wound healing and cause insulin resistance in high-fat diet-fed mice by modulating macrophage phenotypes 21, 22. Furthermore, studies in diabetic patients shows deficient SIRT6 expression in many human tissues, suggesting that SIRT6 has a protective effect on diabetes-associated diseases 20, 23, 24. Therefore, exploring the underlying mechanism of SIRT6 in the regulation of immune homeostasis might have an important clinical significance for achieving good periodontium preservation in DP patients.

Here, we demonstrate that the accumulated apoptotic neutrophils and larger amounts of NET release are mainly responsible for the prolonged inflammation in DP. By constructing myeloid-specific knockout mice and exploring the underlying regulatory mechanism, we discover that the SIRT6-miR-216/217 axis potently modulates the macrophage efferocytosis to clear the apoptotic neutrophil through targeting efferocytosis-related molecules DEL-1 and CD36. Therapeutically, local inhibition of miR-217 in DP models contributes to reduced apoptotic neutrophil accumulation, diminished inflammatory response, and retained periodontium, which might represent an effective strategy for diabetes-associated diseases.

## Materials and methods

### Specimen collection and preparation

The human subject protocol was approved by the Ethical Committee Department at Affiliated Hospital of Stomatology of Nanjing Medical University (Approval No. PJ2020-126-001). The gingival specimens in this study were mainly obtained from the gingivectomy during crown lengthening or immediate implantation or extraction of consecutive multiple teeth, and gingival resection during surgery for a benign cyst of the jaw in the surgical ward, and all participants had informed consent. According to the new classification of the 2018 periodontal workshop^25^, CP inclusion criteria include: 1) patients diagnosed with periodontitis without treatments in the last 6 months, 2) with normal blood glucose without diabetes mellitus, 3) presenting at least 4 teeth with a probing depth ≥6 mm, 4) clinical attachment loss ≥ 3 mm, 5) bleeding on probing index ≥2, and 6) obvious bone loss under radiographic examination. DP inclusion criteria include periodontitis patients diagnosed with T2DM for more than six months and in stable condition. The other criteria include: 1) no cigarette smoking, 2) no systemic diseases, 3) no anti-inflammatory medication intake during the past 3 months, 4) no periodontal treatment during the past 6 months, 5) no pregnant or breastfeeding, 6) no acute infections or allergies, and 7) no antibiotics or immunosuppressant or diabetes mellitus-related drugs treatment in the past three months. Clinical data of the patient are provided (Material S2). The gingival tissue of five diabetic periodontitis and five periodontitis biopsies were collected and then fixed in 4% paraformaldehyde solution for 24 h. After that, the samples were dehydrated, embedded in paraffin, and sectioned into 4 μm slices.

### 2.2 Experimental animals

All animal experimental procedures were approved by the Laboratory Animal Care and Use Committee at Nanjing Medical University (Approval No. IACUC-2004031). Mice were housed under specific pathogen-free conditions on a standard 12/12 h light/dark cycle. Male wild-type C57BL/6 mice (6-8 weeks) were used in this study. The mice were randomly divided into the normal chow diet (ND) group and the high-fat diet (HFD) group (60% kcal fat; Research Diets, Inc), with 5 mice in each group. After 4 weeks, HFD-fed mice were injected with streptozotocin (STZ, 35 mg/kg, Sigma-Aldrich) intraperitoneally for 3 days (once a day) while ND-fed mice were injected with citrate buffer solution. Evaluation criteria for the successful establishment of the T2DM mice model included: 1) Fasting glucose of mice was greater than 16.7 mM. 2) Increased food and water intake, boosted urine output, and lost weight (Figure S2 A-B). The SIRT6^flox/flox^ mice and LysM-Cre Mice were obtained from the Model Animal Research Center of Nanjing University. SIRT6^flox/flox^ and homozygous LysM-Cre mice were crossed to obtain LysM-Cre+ SIRT6^flox/flox^ mice (mS6KO). In the obtained mS6KO mice, only males were used for this experiment.

### Resolving and non-resolving model of periodontitis in mice

In this study, four kinds of mice were used to induce resolving and non-resolving models of periodontitis: 1) ND mice, 2) HFD mice, 3) mS6KO, 4) and mS6KO littermate wild-type mice (WT). As for the non-resolving model of periodontitis, a 4-0 silk ligature was tied around the maxillary left second molar of mice. The contralateral molar tooth in each mouse was left non-ligated to serve as a baseline control for bone loss measurements. The ligatures remained in place for 14d (14DL group). In the resolving model of periodontitis, ligatures were removed on day 7 to allow the transition to inflammation resolution, and the mice were monitored for another 7 d (7DL-7DR group) 16. After the model induction, the mice were euthanized and the maxillary bones were harvested for micro-CT and histological analysis. Maxillary bones were excised and immediately fixed in a 4% paraformaldehyde neutral buffer solution for 48 h. Then, the maxillary specimens were decalcified with 20% EDTA at 4 °C for 30 days until the alveolar bone could be easily penetrated followed by conventional dehydration and paraffin embedding. For CT and histochemical detection, we selected the proximal and distal parts of ligated left second molars as the measurement data.

### Hematoxylin and Eosin, Masson stain and Immunofluorescence Staining

Hematoxylin and eosin (HE), Masson stain, and immunofluorescence staining were processed according to our published work 26. For immunofluorescence staining, all antibodies and TUNEL staining kits according to the instructions (Table S1). NET (MPO^+^ H3cit^+^), M1 (CD68^+^ CD86^+^/ F4/80^+^ CD86^+^), M2 (CD68^+^ CD206^+^/ F4/80^+^ CD206^+^), apoptotic cells (TUNEL positive), and neutrophils (Ly6g^+^ or CD15^+^) cells were determined using ImageJ 1.51r (NIH). The percentage of NET, M1, M2, apoptotic cells, and neutrophils were calculated by dividing the total number of cells (DAPI nuclear staining) of the section to get the percentage of total cells in each field. ImagePro Plus 7.0 software was used to determine the expression of DE-L, CD36, and SIRT6.

### Cell isolation, cell lines, and culture

Peripheral blood neutrophils were collected from healthy donors or mice using a peripheral blood neutrophil separation kit (TBD; Tianjin, China). Briefly, neutrophils were isolated by erythrocyte sedimentation and centrifugation according to the manufacturer's instructions. Blood was separated into plasma, monocytes, neutrophils; and red blood cells from top to the bottom. The layer of neutrophils was carefully transferred into new tubes to lyse red blood cells. Then, cells were washed twice with phosphate-buffered saline (PBS) and centrifuged at 350 g for 10 min. Neutrophils were cultured overnight to induce apoptosis in PBS containing 1% FBS.

Two types of macrophages were used in this study: macrophages induced by human monocytes (THP-1 cells) and macrophages induced by mouse BMDMs (bone marrow-derived macrophages). Human THP-1 acute monocytic leukemia cells were maintained in an RPMI-1640 medium (Gibco, Waltham, MA, USA) containing 10% heat-inactivated fetal bovine serum (FBS, Gibco, Waltham, MA, USA), 1% penicillin/streptomycin mixed solution. THP-1 monocytes were differentiated into M0 macrophages by 48-h incubation with 80 ng/mL PMA (Sigma, Munich, Germany). Mouse bone marrow cells were flushed out of the bone marrow cavities of the femurs and tibias with DMEM containing 2% fetal bovine serum (FBS) and antibiotics (100 U/mL penicillin and 100 mg/mL streptomycin; GE Healthcare, Chicago, IL, USA). The collected cells were cultured in complete DMEM supplemented with 10% FBS, 100 U/mL penicillin, and 100 mg/mL streptomycin and murine M-CSF (10 ng/mL) for 3 d and changed with a fresh medium on the third day. They were continually cultured for an additional 4 d to obtain the mature mouse macrophages.

### Phagocytosis of apoptotic cells

Efferocytosis was assessed in macrophages with autologous apoptotic neutrophils and quantified by flow cytometry. *In vitro* macrophage phagocytosis assays, for fluorescence microscopy, macrophages were labeled with CFSE, and apoptotic neutrophils were labeled with DIL. For flow cytometry, macrophages were labeled with CD68 or F4/80 while apoptotic neutrophils were labeled with CFSE. Subsequently, human or mouse macrophages were co-cultured with apoptotic neutrophils of human or mouse origin at the ratio of 5:1 of apoptotic neutrophils to macrophages for 45 min. Macrophages were then washed three times with PBS and fixed with 4% paraformaldehyde for 15 min to evaluate efferocytosis by microscopy or flow cytometry. For microscopic analysis, at least three different fields were analyzed for each sample, and at least 30 macrophages were evaluated for each field.

### RNA pull-down and RNA-binding protein immunoprecipitation (RIP) assays

Sense and antisense pri-miR-217 were subjected to RNA pull-down assays. The sequence of the pri-miR-217 probe for RNA pull-down assays was 5′-CTTCTTGCCA CATCTTTCAGGTTTCTA-3′, and the antisense was 5′-TAGAAACCTGAAAGATGTGGCAAGAAG-3′. Pierce™ Magnetic RNA-Protein Pull-Down Kit (Thermo, USA) was used to enrich pri-miR-217 binding protein according to the guideline. The enriched proteins were analyzed by silver staining and WB.

According to the manufacturer's instructions, the magnetic RNA-Protein Pull-Down Kit (Thermo, USA) was used to enrich hnRNPA2B1 binding RNA. The enriched RNA was isolated and then subjected to qRT-PCR. 2^ -ΔCT^ was calculated and normalized to the 2^-ΔCT^ of 10% input.

### Chromatin immunoprecipitation (ChIP) assays

Magna ChIP^®^ Protein G Magnetic Beads (Millipore, USA) for use in chromatin immunoprecipitations (ChIP assays). Macrophages were fixed with 1% formaldehyde and then collected by scraping with lysis. Nuclei were then sonicated to break the DNA into a 300 bp fragment. Fragmented soluble chromatin was immunoprecipitated with anti-H3K56ac and Protein A/G. The enriched DNA was isolated by incubating with proteinase K and RNase A. Successful enrichment of H3K56ac-associated DNA fragments was then analyzed by qPCR. Fold enrichment reflects the ratio of H3K56ac signals to that of IGg signals derived from a standard curve of input DNA in qPCR. The information on primers was listed (Table S2).

### Fluorescent *in situ* hybridization (FISH)

All FISH probes were designed and synthesized by RiboBio (Guangdong, China). The FISH assay was performed to detect the location of pri-miR-217 in macrophages on coverslips according to the manufacturer's instructions. DNA was stained with DAPI for 3 min before sealing.

### Co-immunoprecipitation experiment (Co-IP)

Cells were lysed with immunoprecipitation lysis buffer (Beyotime Biotechnology, Shanghai, China) on ice for 30min. The supernatants were incubated overnight at 4 °C with anti-hnRNPA2B1, anti-DGCR8, and anti-Drosha with shaking followed by 4 h of incubation with protein A agarose beads. Subsequently, the samples were washed with immunoprecipitation buffer three times and boiled for 5 min.

### Transfection and dual-luciferase reporter assay

293T cells were seeded in 24-well plates. After 24 h, a scrambled miRNA control and has-miR-216a-5p, has-miR-216b-5p and has-miR-217-5p mimics or has-miR-216a-5p, has-miR-216b-5p and has-miR-217-5p inhibitors (RiboBi, Guangdong, China) were transcribed by using Lipofectamine 2000 (Invitrogen). Dual-Luciferase Reporter Assay System (Promega, Madison, WI, USA) was used to measure luciferase activities after 48h transfection. Firefly luciferase activity of each sample was normalized to renilla luciferase activity.

### Statistical Analysis

The data are presented as the means ± SEM. Each experiment was repeated at least three times independently. The comparisons between the two groups were performed with Student's t-test. Multiple comparisons were assessed by one-way ANOVA. A rank-sum test was used for rank data. p < 0.05 (*), p < 0.01 (**) and p < 0.001 (***) was considered statistically significant.

## Results

### The characteristic distribution of neutrophils and macrophages in human DP

**Figure 1:**
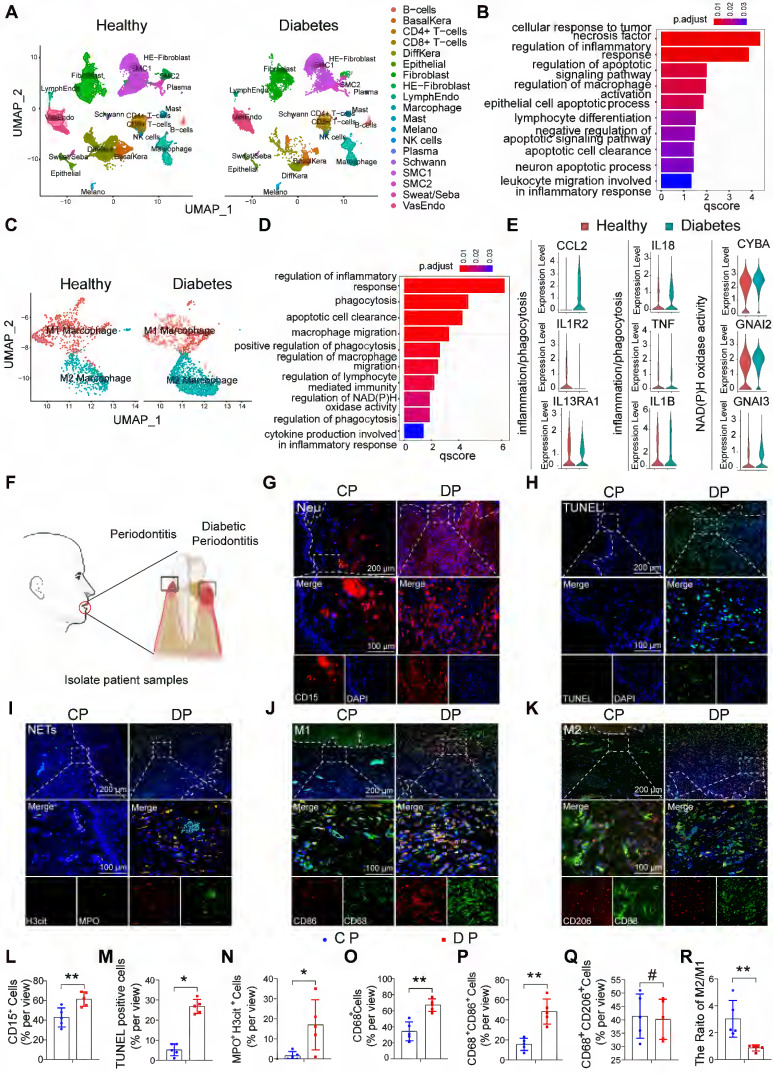
** The characteristic distribution of neutrophils and macrophages in human DP. (A)** Cluster analysis using the Uniform Manifold Approximation and Projection (UMAP) technique of single-cell sequencing from healthy non-DM and Diabetic foot ulceration non-healers skin samples revealed 19 distinct cell clusters. **(B)** GO biological process enrichment of all distinct cells in healthy and DFU-Non-healer. **(C)** UMAP of macrophage clusters, annotated and colored by the sample type and clustering. **(D)** GO biological process enrichment of macrophages in healthy and DFU-Non-healer. **(E)** Violin plots showing expression levels of key upstream regulators in phagocytic, inflammation, and NAD metabolism-related pathways in macrophage clusters. **(F)** Schematic diagram of clinical sample collection. **(G)** Neutrophils were identified by CD15(red) in gingivae. **(H)** Representative images of TUNEL staining in each group and observed under a fluorescence microscope (the nucleus was blue, and the apoptotic cell nucleus appeared green), and the percentages of apoptotic cells were quantitatively analyzed. **(I)** NETs were identified by Cit-H3 (red), MPO (green), and DAPI (blue) in the gingivae of periodontitis and those with diabetic periodontitis. **(J-K)** Immunofluorescence staining of gingival tissues, in which CD68 positive represents macrophage, CD86 positive represents M1 phenotype macrophage, and CD206 positive represents M2 phenotype macrophage. **(L-R)** Quantification of neutrophils, TUNEL positive apoptotic cells, NET, macrophage infiltration, and M1 and M2 polarization. The results were presented as means ± S.D. *p < 0.05; **p < 0.01; #p > 0.05 by 2-tailed, unpaired Student's t test. CP: chronic periodontitis, DP: diabetic periodontitis. The white dotted line indicates the gingival epithelial basement membrane.

To explore differences in cell population composition and regulatory cell pathways in diabetes-related inflammatory diseases, we analyzed foot skin samples from healthy non-DM and diabetic foot ulceration non-healers (DFU-Non-healer) in the single-cell sequencing dataset GSE165816 (Figure S1A) 27. The results of GO enrichment analysis showed that inflammation and apoptosis-related pathways were activated (Figure 1A, B). Among the inflammatory cells identified, we focused on increased macrophage infiltration and further identified the cell populations of the macrophage cluster by GO analysis and UMAP analyses. Notably, UMAP analyses showed an abnormal polarization of macrophages (Figure 1C). GO enrichment analysis revealed abnormalities in pathways related to immune response, phagocytosis, migratory capacity, and glucose metabolism in diabetic macrophages, compared to non-DM control macrophages (Figure 1D, E). These results indicate that the abnormal inflammatory response in diabetic wound healing may be caused by the massive activation of apoptosis-related pathways and the abnormal phagocytic ability of macrophages.

To further explore whether persistently elevated inflammation in DP is associated with abnormal macrophage phagocytic capacity, we collected gingival samples from periodontitis patients with or without diabetes (Figure 1F). The gingival tissues of DP exhibited significantly higher amounts of inflammatory cell accumulation compared with those in CP (Figure S1B). In periodontitis, neutrophil efferocytosis by macrophages is considered an anti-inflammatory and pro-resolving event 10, 16. Chronic inflammation in diabetes is often severe and difficult to resolve. Therefore, we first detected neutrophils in the gingiva, and the results showed that the number of neutrophil-infiltrating (CD15^+^) cells in DP was higher than that in the CP group (Figure 1G, L). Serial sections of gingival tissue were used for TUNEL staining, and the results showed a large number of apoptotic cells in the gingival tissues of DP while almost no visible apoptotic cells in CP (Figure 1H, M). In addition, we found that most apoptotic cells were CD15^+^ neutrophils by comparative observation of serial sections at the same sites (Figure 1G, H). Subsequently, using MPO and H3cit marker NETs, we found that the excessive accumulation and delayed removal of neutrophils in DP led to the formation of a large number of NETs (Figure 1I, N) which consequently aggravated inflammation in diabetic patients and delayed the resolution of inflammation. To further explore whether the apoptotic neutrophils that were not cleared in time were caused by the abnormality of macrophage infiltration, we performed immunofluorescence staining of macrophage polarization. The infiltration of macrophages (CD68^+^) in the gingival of DP is much higher than that of CP (Figure 1O). The infiltration of CD68 CD86-positive M1 macrophages in DP was higher than that in the CP group (Figure 1J, P), and there is no difference between DP group and CP group in CD68 CD206 positive M2 polarized macrophages (Figure 1K, Q). However, the M2/M1 ratio in the DP group was significantly lower than that in the CP group (Figure 1R). In conclusion, the excessive accumulation and delayed clearance of apoptotic neutrophils and NETs, accompanied by increased macrophages infiltration and disrupted macrophage polarization, undoubtedly aggravate DP inflammation.

### Dysfunctional neutrophils and macrophages aggravate inflammatory damage and impair inflammation resolution in mice DP

**Figure 2:**
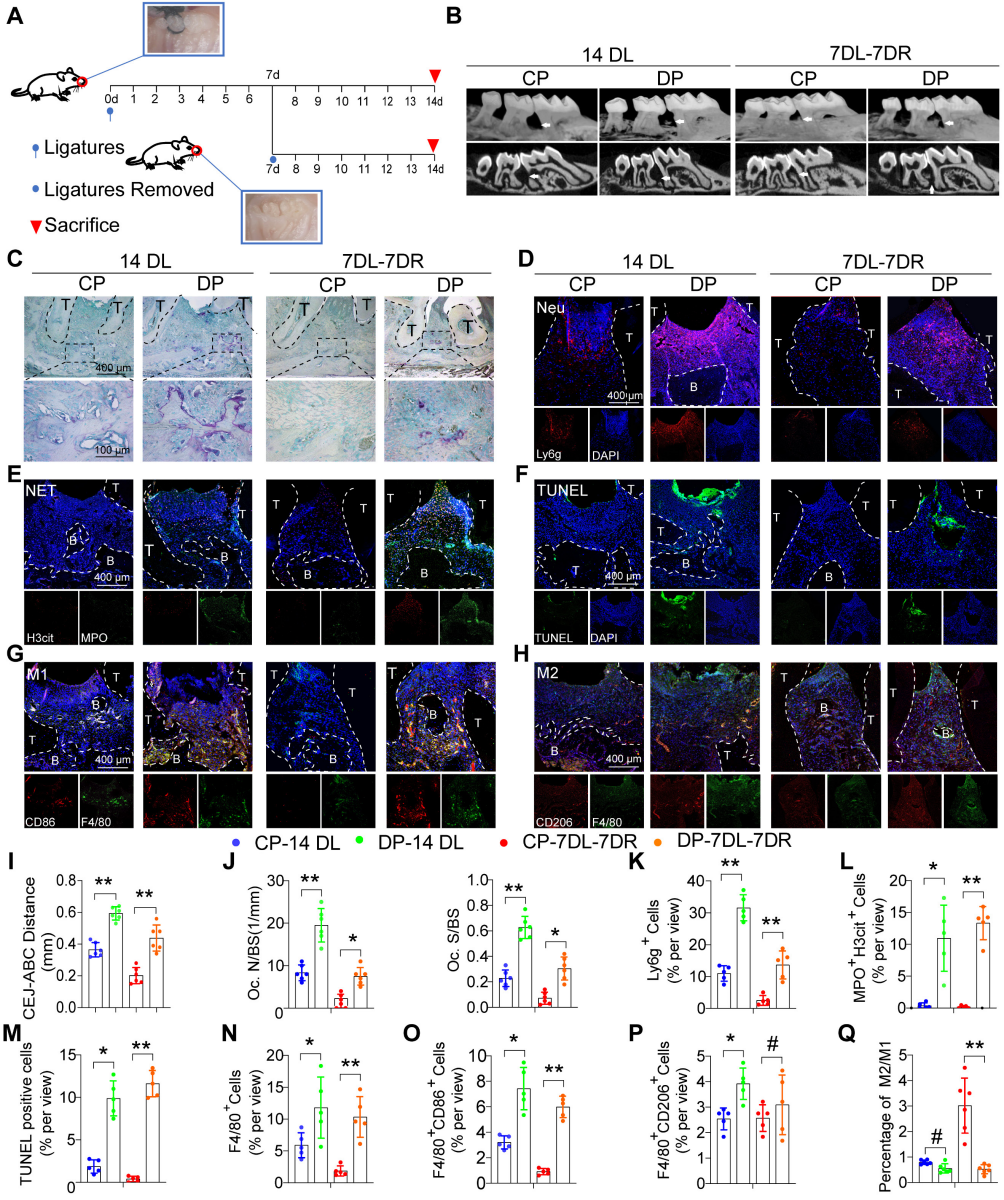
** Dysfunctional neutrophils and macrophages aggravate inflammatory damage and impair inflammation resolution in mice DP. (A)** Schematic diagram showing the induction of the LIP (ligature-induced periodontitis) and LIP resolution models. **(B)** Representative 3D micro-CT scanning images and reconstructed sections (longitudinal direction of the maxillae). The distance of the Cemento-Enamel Junction (CEJ) to the Alveolar Bone Crest (ABC) in mm was analyzed. Arrowhead: the area of loss of alveolar bone. **(C)** Representative images of TRAP-stained paraffin sections. OC. N/ BS (osteoclast number per bone perimeter) and OC. S/BS alveolar bone surface covered by TRAP-positive osteoclasts) were used for quantitative analysis. **(D)** There are representative images of Ly6g positive neutrophils in the periodontium of control and diabetic LIP resolution mice. **(E)** Representative images of Cit-H3 (red) and MPO (green) positive NETs in the periodontium of control and diabetic LIP resolution mice. **(F)** Representative images of TUNEL staining. **(G-H)** Immunofluorescence staining of the periodontium, in which CD68 (green) positive represents macrophage, CD86 (red) positive represents M1 phenotype macrophage, and CD206 (red) positive represents M2 phenotype macrophage. **(I-Q)** Quantification of the distance of CEJ-ABC, OC. N/ BS, OC. S/BS, neutrophils, NET, TUNEL positive apoptotic cells, macrophage infiltration, and M1 and M2 polarization (n = 5 mice in the CP group and n = 5 mice in the DP groups). The results were presented as means ± S.D. *p < 0.05; **p < 0.01; #p > 0.05 by 2-tailed, unpaired Student's t test. T: Tooth, B: Bone of Alveolar, CP: chronic periodontitis, DP: diabetic periodontitis, 14DL: 14 days ligated; 7DL-7DR: 7 days ligated and 7 days with ligatures removed. The white dotted line indicates the boundary between the root and the alveolar bone and the gingiva.

To explore the impact of diabetes on periodontitis progress and inflammation resolution, we used two classic ligature-induced periodontitis (LIP) mice models, namely, the periodontitis model and the periodontitis resolution model. The ligature process was conducted for up to 14 days in the periodontitis model while the mice in the periodontitis resolution model were ligatured for 7 days and then removed ligatures to simulate the course of the inflammation resolution for another 7 days (Figure 2A). Micro-CT and histomorphometric analyses were performed to observe the alveolar bone loss and inflammation (Figure S2C, S2D). The diabetic mice exhibited a marked increase in the distance from the CEJ to the ABC in both the periodontitis and periodontitis resolution models (Figure 2B, I). The diabetic mice displayed more osteoclast activity with higher TRAP-positive osteoclast surface occurring compared with the CP group in both the periodontitis and periodontitis resolution models (Figure 2C, J). In non-resolving models of periodontitis, a large number of neutrophils and macrophages infiltrate into the gingival tissue, after the removal of ligation, neutrophil and macrophage infiltration was reduced (Figure S2E). In addition, large numbers of neutrophil infiltration and MPO^+^ and H3cit^+^ NETs were observed in the periodontium of DP (Figure 2D, K, E, L). TUNEL staining also showed that there were a large number of apoptotic cells in the periodontium of DP while no visible apoptotic cells were observed in the CP group (Figure 2F, M). The numbers of F4/80^+^ macrophages infiltration in DP were greater than that in the CP group (Figure 2N). Concretely, the number of F4/80^+^ CD86^+^ M1 macrophages (Figure 2G, O) and F4/80^+^ CD206^+^ M2 macrophages (Figure 2H, P) in DP was significantly higher than in the CP group. However, the ratio of M2/M1 macrophages was also significantly lower in DP compared to the CP group in the resolution of the LIP model (Figure 2Q). Collectively, the accumulation of apoptotic neutrophils and NETosis in the periodontal tissue accelerated periodontitis progression, and the delayed clearance of apoptotic neutrophils and NETs impeded resolution of inflammation in mice DP.

### SIRT6 dramatically regulates macrophage efferocytosis under high glucose conditions

**Figure 3:**
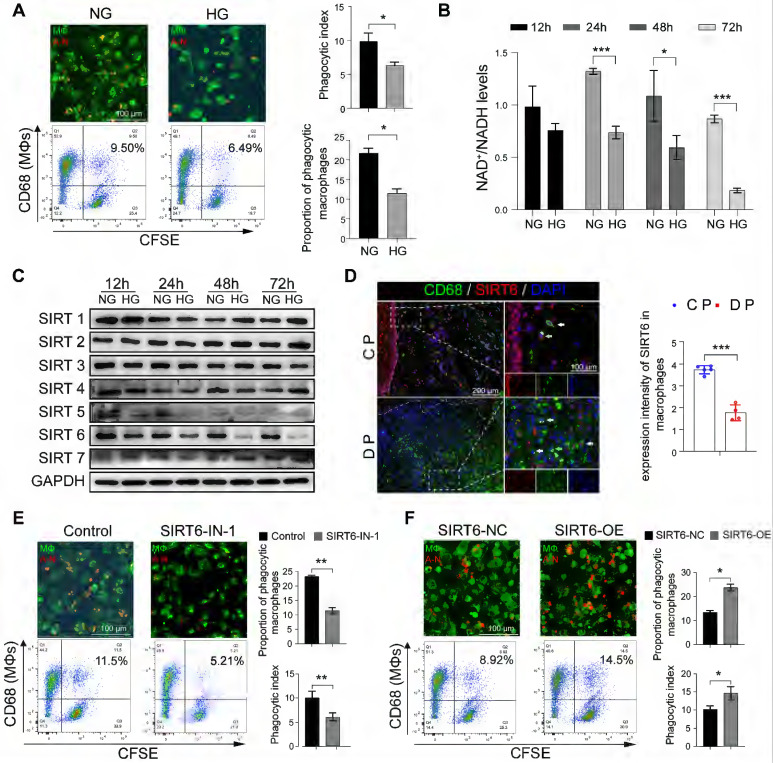
** SIRT6 dramatically regulates macrophage efferocytosis under high glucose conditions. (A)** The phagocytic index was assessed by Immunofluorescence and flow cytometry. Efferocytosis was assessed in macrophages induced by THP-1 with CFSE-labelled autologous cell-tracker (red)-tagged apoptotic neutrophils **(B)** Mitochondrial NAD^+^/NADH ratio detected by NAD^+^/NADH Assay Kit with WST-8. **(C)** The expression of Sirtuins in macrophages after High glucose stimulation. **(D)** Co-staining of CD68, as a marker for macrophages, and SIRT6 in the gingivae of periodontitis and those with diabetic periodontitis **(E-F)** Phagocytic index of macrophages after SIRT6 inhibit and overexpression. The results were presented as means ± S.D. *p < 0.05; **p < 0.01; ***p > 0.001 by ANOVA or Student's t test.

In view of the above results of increased macrophages and apoptotic neutrophils in the gingiva of DP, we further investigated and found that high glucose (HG) did not increase the spontaneous apoptosis of neutrophils *in vitro* (Figure S3A, S3B). Therefore, these results prompted us to decipher whether HG inhibited the efferocytosis of macrophages on apoptotic neutrophils and then indirectly increased their residue. The ability of macrophages to phagocytize apoptotic neutrophils decreased after HG stimulation (Figure 3A). Given that GO enrichment analysis of macrophage clusters in DM showed abnormal NAD^+^ metabolism (Figure 1D, E), we further examined and found that HG stimulation can lead to abnormal NAD^+^/NADH metabolism (Figure 3B). Sirtuins (SIRTs) are a family of NAD^+^ dependent histone deacetylases that takes play in glucose homeostasis, inflammation, genomic stability, and DNA repair 18, 19. Thus, we identified and focused on SIRT6 on account of its obvious downregulation under the HG condition, which was most obvious at 48 h and 72 h (Figure 3C). To further investigate the possible functions of the SIRT6 in the macrophages in DP, co-staining of CD68 (a marker for macrophages) and SIRT6 show that SIRT6 expression in macrophages from DP significantly decreased compared with CP (Figure 3D). SIRT6 inhibitor significantly reduced the capacity of macrophages carrying out efferocytosis while SIRT6 overexpression improve its ability to phagocytize apoptotic neutrophils (Figure 3E, F). Transwell and scratch experiments were used to test the effects of efferocytosis, and apoptotic neutrophils on macrophage migration (Figure S3C, S3D). Compared with efferocytosis, apoptotic cells increase the migration ability of macrophages. Efferocytosis promotes macrophages to secrete anti-inflammatory mediators, such as IL-10 and TGF-β (Figure S3E), while macrophages co-cultured with apoptotic neutrophils secrete a large number of inflammatory mediators, such as IL-6 and TNF-α (Figure S3F). Compared with the control group, the phenotype of macrophages tended to be anti-inflammatory M2 macrophages after efferocytosis, while macrophages co-cultured with apoptotic cells differentiated to M1 and M2, but differentiated more to M1 (Figure S3G, S3H). Based on the above findings, high glucose stimulation leads to low expression of SIRT6 in macrophages, which impairs efferocytosis. Efferocytosis suppresses inflammation by reducing DAMP release from dead cells and maintaining biological homeostasis. However, when the efferocytosis of macrophages in diabetic patients is impaired, the pro-inflammatory lytic cell deaths including NETosis and necroptosis, through the damage-related molecular recognition model, lead to an increase in macrophage infiltration and breaks the balance between M1 and M2 differentiation.

### Myeloid-specific SIRT6 knockout aggravates periodontitis and impairs inflammation resolution

**Figure 4:**
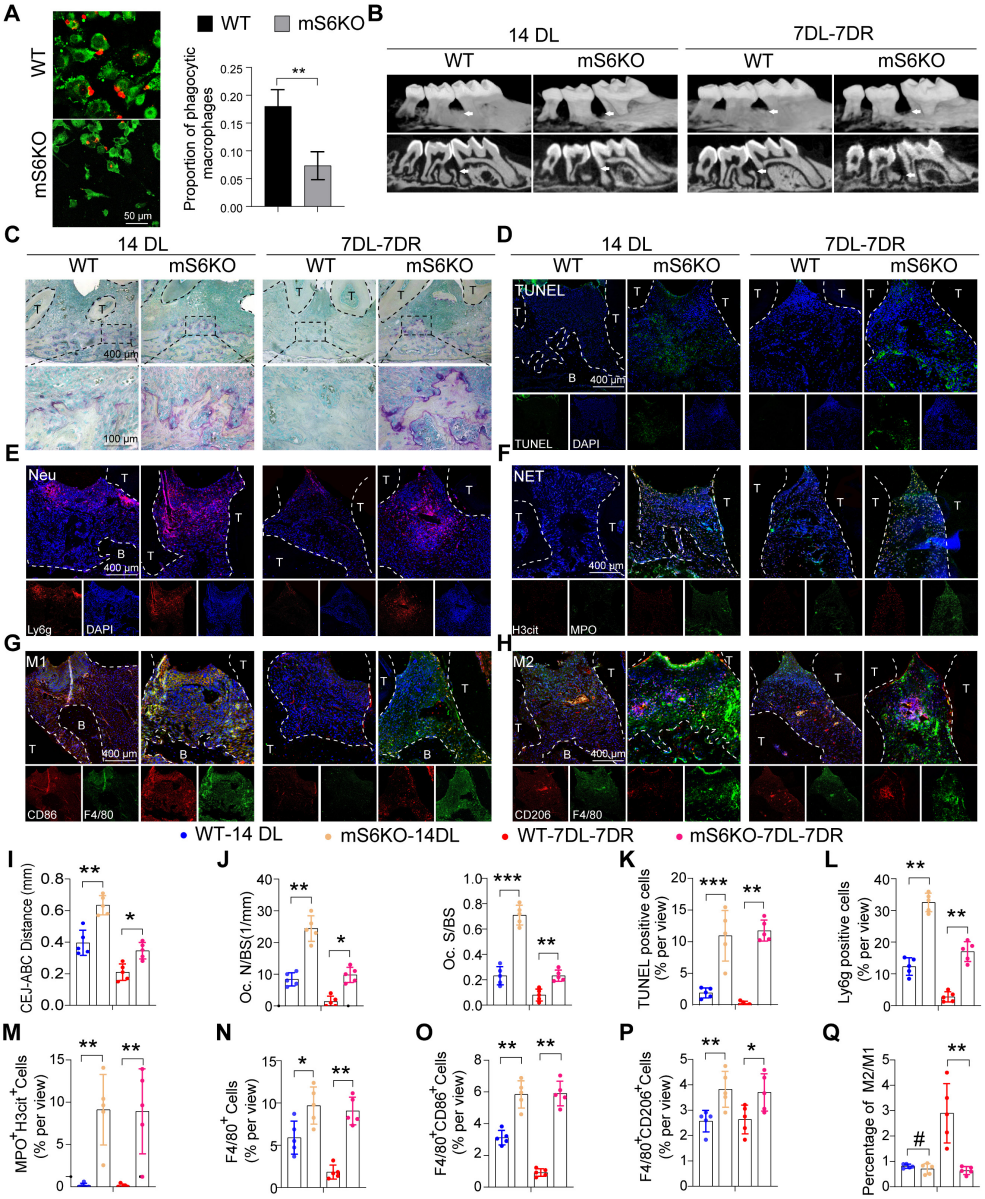
** Myeloid-specific SIRT6 knockout aggravates periodontitis and impairs inflammation resolution. (A)** Phagocytic index was assessed in macrophages of myeloid-specific SIRT6 deficiency mice. **(B)** Representative 3D micro-CT scanning images and reconstructed sections in LIP and LIP resolution of WT and mS6KO mice. Arrowhead: the area of loss of alveolar bone. **(C)** Representative images of TRAP-stained paraffin sections in the periodontium of WT and mS6KO mice. **(D)** Representative images of TUNEL staining in the periodontium of WT and mS6KO mice.** (E)** Representative images of Ly6g positive neutrophils in the periodontium of WT and mS6KO mice. **(F)** Representative images of Cit-H3 (red) and MPO (green) positive NETs in the periodontium of WT and mS6KO mice. **(G-H)** Representative images of M1 and M2 in the periodontium of WT and mS6KO mice. **(I-Q)** Quantification of the distance of CEJ-ABC, OC. N/ BS and OC. S/BS, TUNEL positive apoptotic cells, neutrophils, NET, macrophage infiltration, and M1 and M2 polarization (n = 5 mice in the WT group and n = 5 mice in the mS6KO groups). The results were presented as means ± S.D. *p < 0.05; **p < 0.01; ***p > 0.001 by 2-tailed, unpaired Student's t test. T: Tooth, B: Bone of Alveolar, 14DL: 14 days ligated, 7DL-7DR: 7 days ligated and 7 days with ligatures removed, mS6KO: LysM-Cre+ SIRT6^flox/flox^. The white dotted line indicates the boundary between the root and the alveolar bone and the gingiva.

To explore the role of SIRT6-mediated macrophage efferocytosis in periodontitis, mS6KO mice were generated by breeding LysM-Cre mice with SIRT6^flox/flox^ mice (Figure S4A-B). Compared with bone marrow macrophages (BMMs) from littermate wild-type mice (WT), mS6KO BMMs showed a decreased ability of macrophages to phagocytize apoptotic neutrophils (Figure 4A). Two-month-old mS6KO mice and their WT littermates were used in LIP models and LIP resolution models. mS6KO mice displayed persistent bone loss and periodontal inflammation similar to that of diabetic mice (Figure S4C, S4D). Compared with WT mice, the distance from CEJ to ABC (Figure 4B, I) and trap-positive osteoclasts in mS6KO mice were significantly increased (Figure 4C, J). To investigate whether impaired macrophage efferocytosis was involved in persistent bone loss in mS6KO mice, the number of apoptotic cells in the periodontium of mS6KO mice and WT mice was evaluated by TUNEL staining (Figure 4D, K). The results showed that the number of Ly6g^+^ cells and apoptotic cells significantly increased in the periodontium of mS6KO mice compared with littermate wild-type mice (Figure 4E, L). SIRT6 knockout promoted MPO^+^ H3cit^+^ NETosis in the periodontium of mS6KO mice (Figure 4F, M). Besides, Both F4/80^+^ CD86^+^ M1 macrophages (Figure 4G, O) and F4/80^+^ CD206^+^ M2 macrophages (Figure 4H, P) significantly increased in the periodontium of mS6KO mice (Figure 4N), but the percentage of anti-inflammatory F4/80^+^ CD206^+^ M2 macrophages was lower in mS6KO mice periodontium compared to WT mice (Figure 4Q). Taken together, these findings indicate that SIRT6-mediated macrophage efferocytosis is associated with periodontal destruction and the resolution of inflammation.

### SIRT6 inhibits transcription of miR-216a-5p-216b-5p-217 cluster through H3K56ac

**Figure 5:**
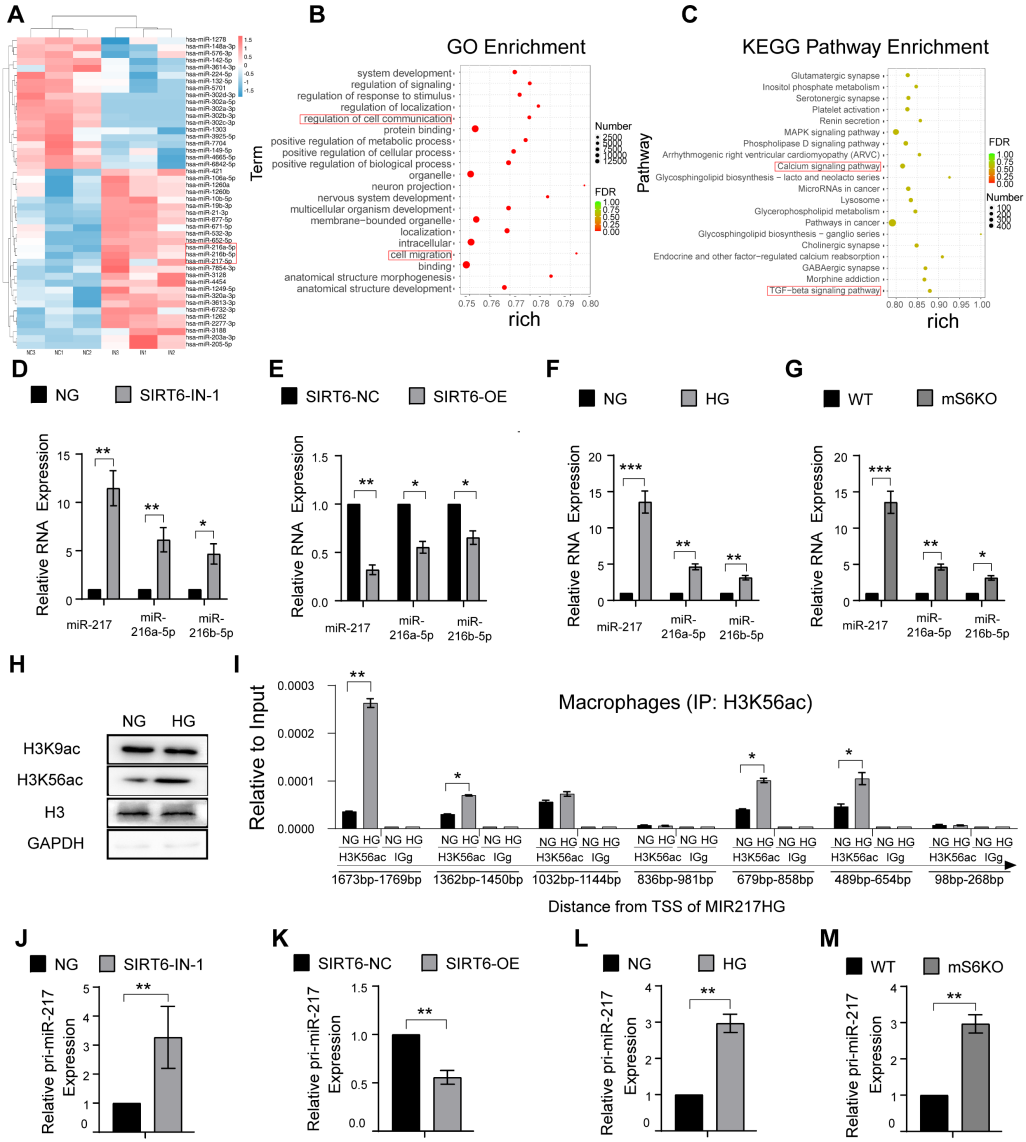
** SIRT6 inhibits transcription of miR-216a-5p-216b-5p-217 cluster through H3K56ac. (A)** miRNA array analysis showed the differential miRNAs in macrophages after SIRT6 inhibition. **(B)** GO enrichment analysis of differential microRNA target genes. **(C)** KEGG Pathway enrichment analysis of differential microRNA target genes. **(D)** The high expression of miR216a-5p-216b-5p-217 cluster after SIRT6 inhibition. **(E)** The low expression of miR216a-5p-216b-5p-217 cluster after SIRT6 overexpression. **(F)** The high expression of miR216a-5p-216b-5p-217 cluster after high glucose stimulation. **(G)** Compared with BMMs of WT mice, Myeloid-specific SIRT6 deficiency mice exhibit high expression of miR216a-5p-216b-5p-217 clusters.** (H)** Western blot revealing the protein expression of H3K9ac and H3K56ac after high glucose stimulation. **(I)** H3K56ac was enriched around the TSS of MIR217HG in macrophages by ChIP-qPCR analysis.** (J)** The high expression of the pri-miR-217 after SIRT6 inhibition. (K) The low expression of pri-miR-217 after SIRT6 overexpression. **(L)** The high expression of the pri-miR-217 after high glucose stimulation. **(M)** Myeloid-specific SIRT6 deficiency mice exhibit high expression of pri-miR-217. The results were presented as means ± S.D. *p < 0.05; **p < 0.01; ***p > 0.001 by 2-tailed, unpaired Student's t test.

To gain insights into the detailed mechanism of how SIRT6 regulates macrophage efferocytosis, the effect of SIRT6 on the expression of microRNA in macrophages was further evaluated. RNA was isolated from macrophages after SIRT6 inhibition for miRNA PCR microarray analysis, and the results showed that miR-216a-5p-216b-5p-217 cluster increase was obvious along with downregulated SIRT6 expression (Figure 5A, B, C). Recent studies have shown that miR-216/217 plays a critical role in age-related diseases including atherosclerosis and diabetes mellitus 28-31. Consistent with these results, our data further showed increased miR-216a-5p-216b-5p-217 cluster expression under SIRT6 inhibition and HG condition (Figure 5D, F), and decreased expression under SIRT6 overexpression (Figure 5E). To further delineate how SIRT6 inhibits miR-216a-5p-216b-5p-217 cluster expression, we detected the histone modifications of its host gene MIR217HG promoter in macrophage by ChIP-qPCR. SIRT6 works as a transcriptional co-suppressor by catalyzing the deacetylation of histone H3 lysine residues acetylated at positions 9 and 56 (H3K9ac and H3K56ac) 32. Interestingly, we found that HG treatment only significantly upregulated the expression of H3K56ac (Figure 5H). Seven pairs of primers that covered the miR-216a-5p-216b-5p-217 promoter were used to detect, and the results showed that HG treatment increased the H3K56ac level of the miR-216a-5p-216b-5p-217 cluster promoter (Figure 5I). We also further confirmed increased pri-miR-217 cluster expression under SIRT6 inhibition and HG condition (Figure 5J, L), and downregulated pri-miR-217 after SIRT6 overexpression (Figure 5K). Notably, BMMs from mS6KO mice exhibited increased pri-miR-217 and miR-216a-5p-216b-5p-217 cluster expression (Figure 5G, M).

### SIRT6 restrains miR-216a-5p-216b-5p-217 cluster maturation through ''non-canonical'' microprocessor complex

**Figure 6:**
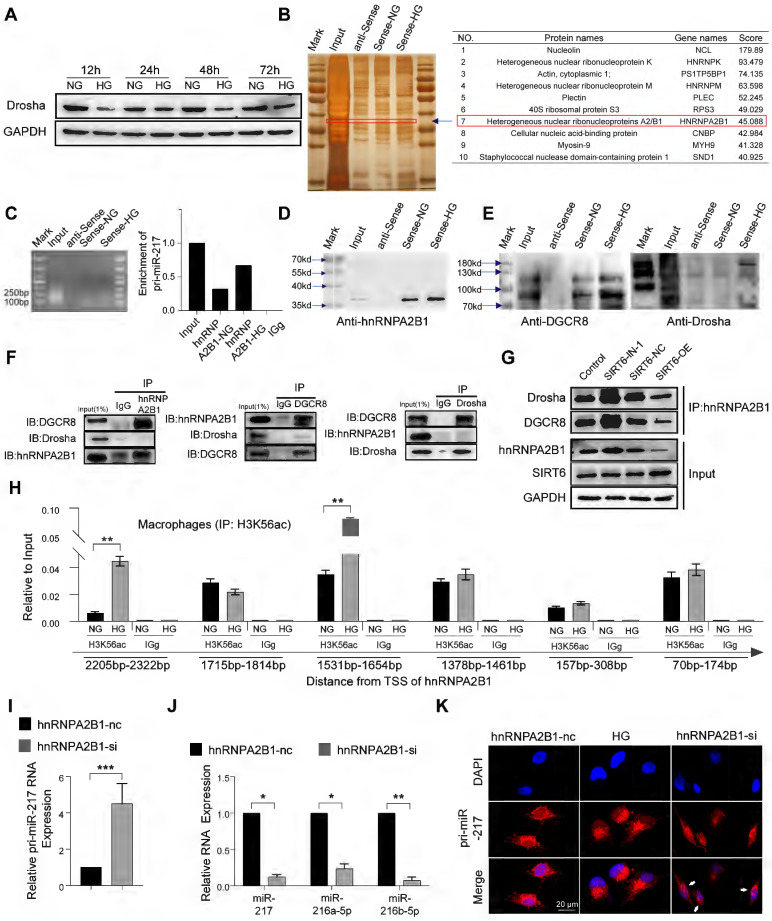
** SIRT6 restrains miR-216a-5p-216b-5p-217 cluster maturation through ''non-canonical'' microprocessor complex. (A)** High glucose stimulation inhibits the expression of the Drosha protein in macrophages. **(B)** Silver staining of pri-miR-217 pulldown in macrophages. List of the top ten differentially expressed proteins identified by mass spectrometry, FDR < 0.05.** (C)** Expression levels of pri-miR-217 were detected by qRT-PCR after RIP for hnRNPA2B1 in macrophages. **(D)** Western blotting showed pri-miR-217 pulldown of the hnRNPA2B1. **(E)** Western blotting showed pri-miR-217 pulldown of the DCGR8 and Drosha. **(F)** Immunoprecipitation of DGCR8 and Drosha and hnRNPA2B1. **(G)** Immunoprecipitation of Drosha and DGCR8 using an anti-hnRNPA2B1 antibody. Inhibition of SIRT6 reduces the formation of a ''non-canonical'' microprocessor complex. By contrast, Overexpression of SIRT6 promotes the formation of a ''non-canonical'' microprocessor complex.** (H)** H3K56ac was enriched around the TSS of hnRNPA2B1 in macrophages by ChIP-qPCR analysis. **(I-J)** The expression of pri-miR-217 and miR-216a-5p/ miR-216a-5p/ miR-217 in macrophages after si-hnRNPA2B1. **(K)** FISH localization of pri-miR-217 in macrophages after si-hnRNPA2B1 or high glucose stimulation. U6 and 18S rRNA were used as positive controls for the nuclear and cytoplasmic fractions, respectively. The results were presented as means ± S.D. *p < 0.05; **p < 0.01; ***p > 0.001 by 2-tailed, unpaired Student's t test.

The canonical microRNA maturation is initially the transcription of a primary microRNA (pri-miR) transcript, which is identified and cleaved by a microprocessor complex composed of ribonuclease III, Drosha, and DGCR8 33, 34. It has been reported that glucose metabolism affects the expression of Drosha protein with glucose deprivation promoting Drosha expression and HG stimulation inhibiting Drosha expression 35. Similarly, our result showed HG stimulation could inhibit the expression of Drosha protein in macrophages (Figure 6A). Despite Drosha decreasing, we found that high glucose resulted in high expression of the miR216a-5p-216b-5p-217 cluster (Figure 5F). To further explore the mechanisms of miR216a-5p-216b-5p-217 clusters maturation under HG conditions, we designed a specific biotin-labeled pri-miR-217 probe to perform an RNA pulldown assay in macrophages, and the silver staining showed enrichment of several bands of proteins combined with pri-miR-217 (Figure 6B). Meanwhile, protein mass spectrometry analysis revealed that hnRNPA2B1 ranked forward in the recognized protein list, and Drosha and DGCR8 were not observed. The RIP assay revealed that antibodies against hnRNPA2B1 pulled down more pri-miR-217 compared to IgG (Figure 6C). hnRNPA2B1 is reported to interact with the microRNA Microprocessor complex protein DGCR8 to cleave primary miRNA through binding to m6A marks primary-miRNA transcripts 36. As expected, the coimmunoprecipitation (Co-IP) of the endogenous hnRNPA2B1 protein and DGCR8 and Drosha in the macrophages indicated their direct physical interaction, which showed hnRNPA2B1 binding to DGCR8 while no Drosha was observed (Figure 6F). We further determined that DGCR8 can bind to Drosha, which means that hnRNPA2B1 binds to DGCR8 to recruit Drosha to form a microprocessor complex, and the ''non-canonical'' microprocessor complex identifies and cleaves pri-miR-217 (Figure 6E). The Co-IP also revealed that SIRT6 inhibition promoted the formation of a ''non-canonical'' microprocessor complex, and SIRT6 overexpression reduced the microprocessor complex (Figure 6G). Besides, we further identified that high glucose not only increased the hnRNPA2B1 bound to pri-miR-217 (Figure 6D) but also increased the expression of hnRNPA2B1 by western blotting analysis in macrophages (Figure S5A). The results of Chip also showed that HG treatment increased the H3K56ac of the hnRNPA2B1 promoter (Figure 6H). To determine whether HG affects pri-miR-217 processing in an hnRNPA2B1-dependent manner, we performed hnRNPA2B1 knockdown to macrophages and found that levels of the mature miRNAs decreased whereas levels of their pri-miR-217 increased (Figure 6I, J). FISH staining showed that HG stimulation led to the decrease of pri-miR-217 in the nucleus, and the accumulation of pri-miR-217 in the nuclei after knockdown of hnRNPA2B1 (Figure 6K). These data indicate that SIRT6 not only promotes the transcription of pri-miR-217 but also promotes the maturation of the miR216a-5p-216b-5p-217 cluster by promoting the formation of ''non-canonical'' microprocessor complex in HG condition.

### miR-216a-5p-216b-5p-217 cluster negatively regulates macrophages efferocytosis by targeting DEL-1 and CD36

**Figure 7:**
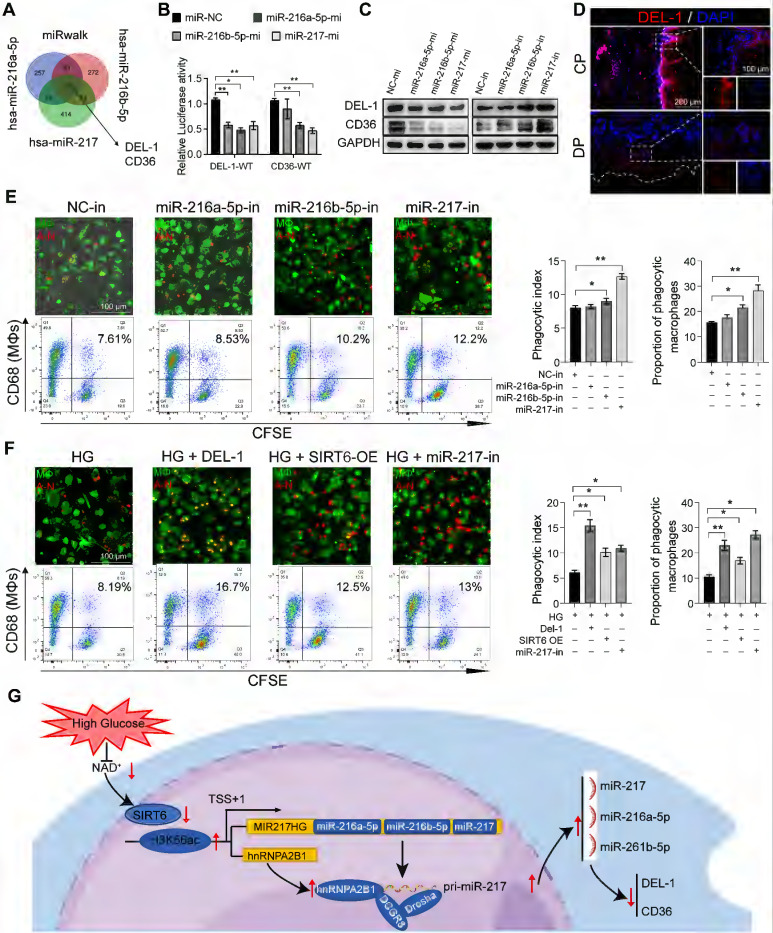
** miR-216a-5p-216b-5p-217 cluster negatively regulates macrophages efferocytosis by targeting DEL-1 and CD36. (A)** Venn diagram showing for miR-217-5p and miR-216a-5p and miR-216b-5p targeted genes predicted by miRwalk. **(B)** Dual-Luciferase Reporter of DEL-1and CD36, and luciferase activity in 293T cells co-transfected with miR-217-5p and miR-216a-5p and miR-216b-5p mimic. **(C)** Western blotting showed the expression of CD36 and DEL-1 following transfected with miR-217-5p and miR-216a-5p and miR-216b-5p mimic and inhibitors. **(D)** The expression of DEL-1 in diabetic periodontitis was decreased compared with periodontitis. **(E)** Phagocytic index of macrophages following exposure to the miR-217-5p and miR-216a-5p and miR-216b-5p inhibitor. **(F)** Under high glucose conditions, exogenous protein DEL-1, miR-217-5p inhibitor and overexpress SIRT6 could restore macrophage efferocytosis. **(G)** Schematic showing the mechanism that SIRT6 mediated macrophage efferocytosis. The results were presented as means ± S.D. *p < 0.05; **p < 0.01 by ANOVA or Student's t test.

DEL-1 and CD36 have been identified as crucial regulatory molecules in macrophage efferocytosis and clearance of inflammation 16, 37. The RT-qPCR results showed that there was no significant change in the RNA level of DEL-1 and CD36 after SIRT6 inhibition, but WB showed low expression (Figure S5B, S5C), so we believed that the regulation mode was post-transcriptional regulation. In view of the above results and the role of miR216a-5p-216b-5p-217 cluster in diabetes 28-31, we hypothesized that SIRT6 regulated macrophage efferocytosis and resolution of inflammation by miR-216/217 cluster/DEL-1 CD36 axis. Consistent with this hypothesis, the results of bioinformatic algorithms (miRwalk) indicated miR-216a-5p-216b-5p-217 cluster binding to the 3'UTR of DEL-1 and CD36 mRNA (Figure 7A). Subsequently, the dual-luciferase reporter assay showed that the co-transfection of the miR216a-5p-216b-5p-217 cluster decreased the luciferase activity of WT-DEL-1-3'UTR and WT-CD36-3'UTR, while the luciferase activity of Mut-DEL-1-3'UTR and Mut-CD36 -3'UTR was not affected (Figure 7B). Furthermore, Western blot analysis exhibited that DEL-1 and CD36 expression were lowered in macrophages treated with miR216a-5p-216b-5p-217 mimic, and DEL-1 and CD36 expression were increased in macrophages treated with miR-216b-5p-217 inhibitor (Figure 7C). Immunofluorescence also confirmed the low expression of DEL-1 and CD36 in diabetic periodontitis (Figure 7D, S6A). Overexpression of the miR216a-5p-216b-5p-217 cluster decreased the phagocytosis of macrophages (Figure 7E), and knockdown increased the phagocytosis of macrophages (Figure S6B, S6C). Among the three microRNAs, miR-217 has the greatest effect on the phagocytosis of macrophages. miR-217 knockdown in an HG environment could restore macrophages efferocytosis like the effects of recombinant DEL-1 protein (Figure 7F). To further confirm the influence of abnormal macrophage efferocytosis in the decreased proportion of M2 macrophages in DP, we restored macrophage efferocytosis by overexpressing SIRT6 or silencing miR-217 in the HG condition and observed the increased polarization of M2 macrophages (Figure S6D, S6E). Overall, our results support the hypothesis that SIRT6 negatively regulates miR216a-5p/216b-5p-217 cluster expression by promoting pri-miR-217 transcription and the formation of pri-miR ''non-canonical'' microprocessor complex. This microRNA cluster inhibits macrophage efferocytosis by targeting the key efferocytosis molecules DEL-1 and CD36, resulting in dysfunctional macrophage-mediated inflammation resolution.

### Local delivery of antagomir-217 promotes resolution of inflammation in mice DP

**Figure 8:**
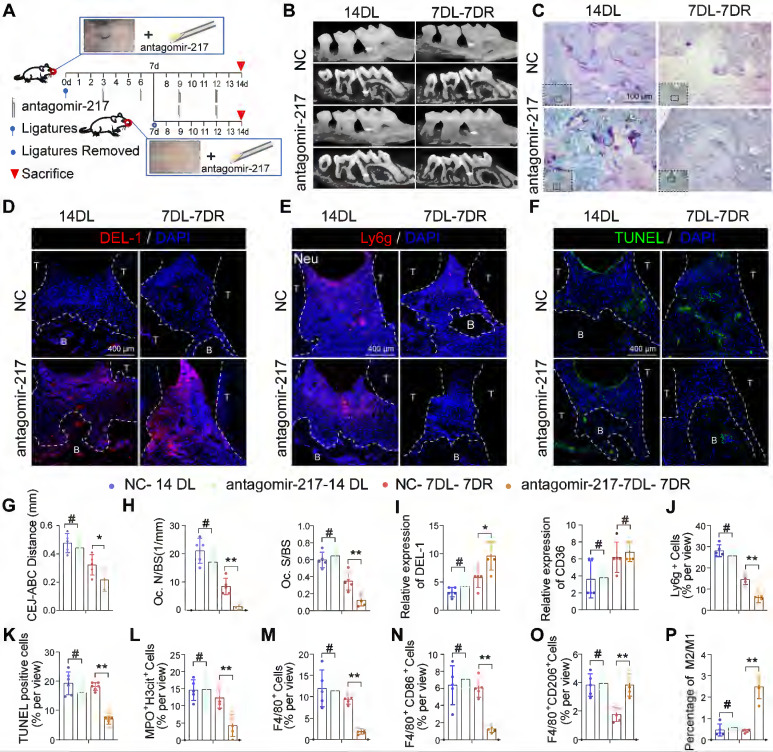
** Local delivery of antagomir-217 promotes the resolution of inflammation in mice DP. (A)** Schematic diagram of the LIP and LIP resolution models and treatment schedule induction. **(B)** Representative 3D micro-CT scanning images and reconstructed sections in LIP and LIP resolution of NC and antagomir-217 treatment mice. Arrowhead: the area of loss of alveolar bone.** (C)** Representative images of TRAP-stained paraffin sections in the periodontium of NC and antagomir-217 treatment mice. **(D)** Representative images of the expression of DEL-1 in the periodontium of NC and antagomir-217 treatment mice. **(E)** Representative images of Ly6g positive neutrophils in the periodontium of NC and antagomir-217 treatment mice. **(F)** Representative images of TUNEL staining in the periodontium of NC and antagomir-217 treatment mice. **(G-P)** Quantification of the distance of CEJ-ABC, OC. N/ BS and OC. S/BS, the expression of DEL-1 and CD36, neutrophils, TUNEL positive apoptotic cells, NET, macrophage infiltration, and M1 and M2 polarization (n = 5 mice in the NC group and n = 5 mice in antagomir-217 groups). The results were presented as means ± S.D. *p < 0.05; **p < 0.01; #p > 0.05 by ANOVA or 2-tailed, unpaired Student's t test. T: Tooth, B: Bone of Alveolar, 14DL: 14 days ligated; 7DL-7DR: 7 days ligated and 7 days with ligatures removed. The white dotted line indicates the boundary between the root and the alveolar bone and the gingiva.

Based on phagocytic index observations in the above gain-of- and loss-of-function studies, miR-217 showed a better phenotype and was selected for *in vivo* intervention treatment. We injected miR-217 specific antagomirs or scramble (NC)-miR into the periodontium of diabetic mice immediately on days 3, 6, 9, and 12 after ligature silk (Figure 8A). As for the LIP model, the injection of antagomir-217 failed to ameliorate diabetic mice's periodontium inflammation and bone loss (Figure S7A, S7B). Intriguingly, antagomiR-217 injection significantly ameliorated diabetic mice periodontium inflammation and bone loss when the ligatures were removed on day 7 (Figure 8B, C, G, H). After antagomiR-217 injection, the expression of DEL-1 in diabetic mice periodontium increased significantly (Figure 8D, I). The number of Ly6g^+^ neutrophils in antagomiR-217 injection diabetic mice periodontium decreased significantly (Figure 8E, J). Compared with antagomir-NC, the formation of MPO^+^ H3cit^+^ NET (Figure 8L, S7D) and apoptotic cells was markedly reduced after injection of antagomiR-217 (Figure 8F, K). A higher percentage of M2 anti-inflammatory macrophages (Figure 8O, S7F) was found in the antagomiR-217 treated group, whereas, in the control group, most macrophages were M1 macrophages (Figure 8M, N, P, S7E). All in all, these results indicate that inhibition of the SIRT6 / miR216a-5p-216b-5p-217 cluster / DEL-1 and CD36 regulatory axis by silencing miR-217 promoted macrophages' efferocytosis to clear apoptotic cells, implying that this regulatory axis has the potential to serve as a target for diabetic inflammation resolution.

## Discussion

Impaired inflammation resolution and persistent inflammatory responses are the typical characteristics in the pathogenesis of diabetic complications 1. Macrophages efferocytosis, an essential process for the inflammation resolution, were functionally impaired in diabetes-associated diseases resulting in a prolonged inflammatory response and relentless tissue damage 4, 14, 17, 38. In this study, we found that impaired efferocytosis and severe periodontal destruction occurred in diabetes-associated periodontitis due to deficient SIRT6 in macrophages. Mechanistically, we demonstrated that HG condition induced SIRT6 downregulation not only resulted in transcriptional activation of pri-miR-217 but also promoted the splicing maturation of the miR216a-5p/216b-5p/217 through the non-canonical pathway by the formation of microprocessor complex. This matured miRNA cluster exploited the inhibitory effect of macrophage efferocytosis by targeting the key efferocytosis molecules DEL-1 and CD36. In addition, we determined the* in vivo* role of the SIRT6-miR-216/217 axis by constructing myeloid-specific deletion of SIRT6 mice and locally delivering antagomir-217 in DP models. Collectively, our data unravel a previously unknown linkage between macrophage efferocytosis and diabetic periodontitis, and targeting the mechanism of the SIRT6-miR-216/217 axis might be a promising strategy for promoting inflammation resolution and restoration of the periodontium.

Macrophages efferocytosis is an aerobic glycolysis-dependent process to clear apoptotic cells 39, suggesting metabolism plays a critical role in immune homeostasis. The NAD^+^ metabolism involving the process of oxidoreductase-mediated hydride transfer is fundamental for various biochemical reactions to execute the function of catabolism and harvesting metabolic energy 40. Noticeably, NAD^+^ metabolic production that is indispensable for the switching from the early to the late stages of acute inflammation potently activated SIRT6 to decrease glycolysis with a low-energy response, obtaining the shift into the late-stage and restoring tissue homeostasis 18. In the context of diabetes, emerging evidence indicates that disrupted NAD^+^ metabolism is increasingly considered the critical factor for the pathogenesis of various diabetes-related diseases 41. Recently, studies show that enhancing the NAD^+^ biosynthesis or reducing its catabolism contributes to the therapeutical effect for diabetic cardiomyopathy 42. In our study, we observed the disrupted NAD^+^ metabolism by GO enrichment analysis and declined ratio of NAD^+^ and NADH in macrophages under HG stimulation, suggesting that the impairment of NAD^+^ metabolism and its involved aerobic glycolysis program might potentially contribute to the dysfunctional macrophages efferocytosis in diabetes. In addition, we here demonstrated that SIRT6, a family of NAD^+^ dependent histone deacetylases, was downregulated in diabetic periodontitis and epigenetically controlled the macrophages efferocytosis by regulating the mature of miR-216/217 and the expression of CD36 and DEL-1. Thus, this important mechanistic insight manifested that SIRT6 might serve as a regulator of a therapeutic target for diabetic periodontitis and related diseases.

Periodontitis that is resulted from oral microbial dysbiosis contributes to oral inflammation and systemic conditions through bacteremia 43. The bacterial diversity and their dynamic balance have been demonstrated to determine periodontal disease activity 44, 45. However, the development of hyperglycemia in diabetic mice triggers a shift in oral bacterial composition. This shift leads to the increasing pathogenicity and accelerated infectious processes that are associated with more severe periodontitis and ultimately tooth loss 46. Notably, the reduced capacity of macrophages to phagocytose pathogens further exacerbates the periodontal inflammation, and declined performance of efferocytosis decreases the number of M2 reparative macrophages 16, 47. Thus, our presented results suggested that restoring the macrophages efferocytosis in diabetic periodontitis not only promotes the clearance of apoptotic cells but also potentially removes the pathogens, strengthening our conclusion that facilitating the function of macrophages efferocytosis is a promising strategy for treating diabetic periodontitis. Further work is needed to elucidate whether the SIRT6-miR-216/217 axis is also involved in the clearance of pathogens, as well as other mechanisms that may contribute to the delayed inflammation resolution of DP.

MicroRNAs (miRNAs) are small non-coding RNAs that are predicted to post-transcriptionally regulate messenger RNA and protein synthesis in mammals 48, 49. The first step of miRNAs biogenesis is the processing of primary microRNAs (pri-miR) by the canonical microprocessor complex composed of ribonuclease III, DROSHA, and DGCR8 50. However, Drosha's splicing of pri-miR is not selective, and its expression and activity itself can be diversely regulated under distinct environments. In diabetic mice, miR-192 biosynthesis could be enhanced via nuclear transfer of YB-1 to recruit Drosha and execute its splicing function 51. The RNA-binding hnRNPA2B1 protein is reported to bind m6A-bearing RNAs to regulate the processing of a subset of pri-miRNAs through interacting with the microprocessor complex protein DGCR8 and Drosha 36. In our study, we found that HG stimulation resulted in low expression of Drosha protein in macrophages, but still increased expression of the miR-216/217 cluster. To resolve this apparent contradiction, we characterized co-precipitated proteins of hnRNPA2B1 and demonstrated that hnRNPA2B1 can directly binds to DGCR8 to recruit Drosha, forming a ''non-canonical'' microprocessor complex. Moreover, we confirmed that hnRNPA2B1 expression was transcriptionally activated due to SIRT6 deficiency in presence of HG condition. All these data suggest a “non-canonical” mechanism of microprocessor complex under SIRT6 modulation occurred in the context of HG for the splicing of pri-miR-217; however, future studies are needed to investigate whether SIRT6-regulated “non-canonical” microprocessor complex participate in the splicing of other pri-microRNA.

Administration of specific miRNA mimics or inhibitors might be promising targets for chronic non-healing wounds 52. In line with the clinical periodontitis, our study showed that local delivery of antagomir-217 significantly ameliorated diabetic mice periodontium inflammation and bone loss in the resolving group, which failed to ameliorate periodontium inflammation in the non-resolving group. Clinically, it is generally difficult to resolve periodontal inflammation via drugs in the context of local irritant dental calculus 5. Similarly, local irritant factor (ligature) might influence the effects of antagomir-217 on the non-resolving group. As mentioned above *in vitro* study, overexpression or inhibition of miR-217 contributed to downregulation or upregulation of DEL1/CD36 expression, respectively. Thus, our data suggested that antagomir-217 promoting inflammation resolution of DP might be attributed to DEL1/CD36-regulated macrophage efferocytosis. However, whether upstream factor SIRT6 have the potentially therapeutical effect for alleviating periodontal inflammation and how to delivery these targeted drugs safely and effectively need to be further investigated.

In conclusion, our findings uncovered the essential role of the SIRT6-miR-216/217 axis in macrophage efferocytosis in the context of diabetes and outlined an approach to improve inflammation resolution and periodontium restoration using inhibition of miR-217. This strategy could be relevant for the management of diabetic diseases and other chronic inflammatory disease.

## Supplementary Material

Supplementary figures, materials and methods, table legends.

Supplementary table 1.

Supplementary table 2.

## Abbreviations

DP: Diabetic Periodontitis
CP: chronic periodontitis
LIP: ligature induced periodontitis
14DL: 14 days ligated
7DL-7DR: 7 days ligated and 7 days with ligatures removed
mS6KO: LysM-Cre+ SIRT6^flox/flox^
