# Supplementary material for: SIRT6-regulated macrophage efferocytosis epigenetically controls inflammation resolution of diabetic periodontitis
Source: Theranostics. 2023 Jan 1;13(1):231–49. doi: 10.7150/thno.78878 (PMC9800730; doi:10.7150/thno.78878)
Supplement: Supplementary file 1 — Supplementary figures, materials and methods, table legends. [file thnov13p0231s1.pdf]

Supplemental Figures

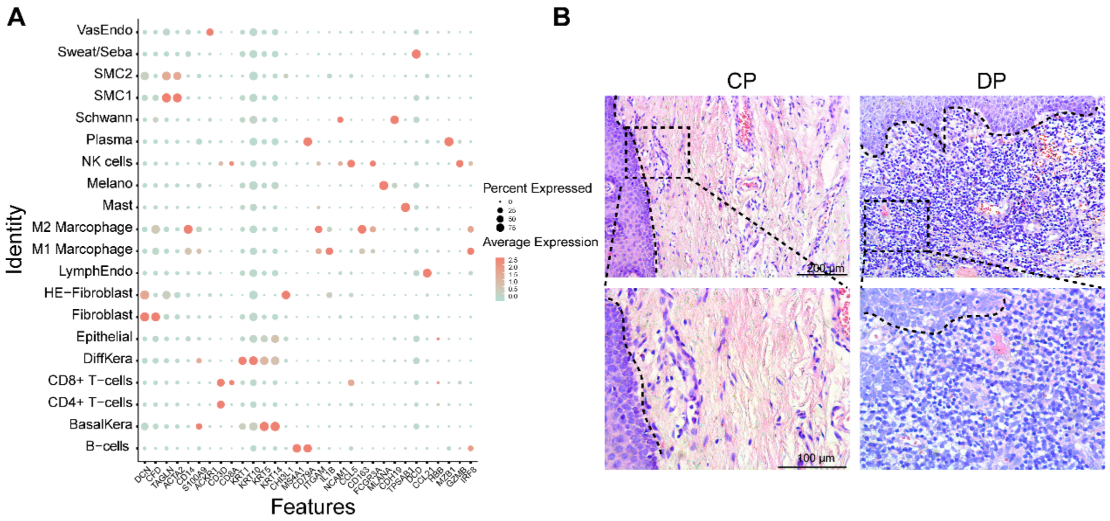

Fig. 1. Dot plots for cell-type annotation and abnormal immune cell accumulation in the gingivae of diabetic periodontitis.

(A) Dot plots showing gene expression of different cell type-specific markers for cell-type annotation. (B) HE staining in the gingivae of periodontitis and those with diabetic periodontitis. The Black dotted line indicates the boundary between the root and the alveolar bone and the gingiva.

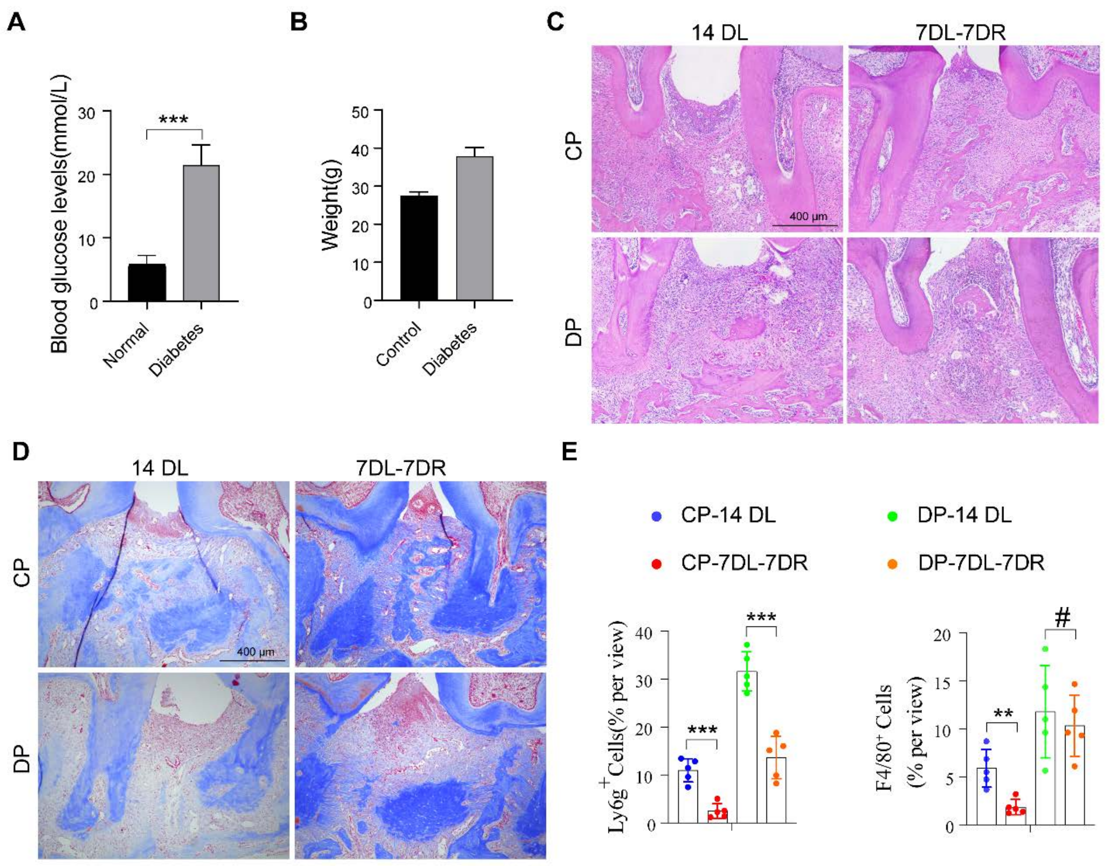

Fig. 2. Diabetes increases alveolar bone loss in ligature-induced periodontitis.

(A) Blood glucose of mice in each group at the beginning of modeling. (B) Weight of WT and diabetes mice at the beginning of modeling. (C) Representative images of H&E-stained paraffin sections. (D) Representative images of Masson-stained paraffin sections. (E) In the LIP group, due to the presence of foreign bodies (ligatures), neutrophils and macrophages infiltrated in large quantities. When ligation was removed, neutrophils and macrophages infiltrated less. The results were presented as means ± S.D. ***$p < 0.001$ by a 2-tailed, unpaired Student's t test.

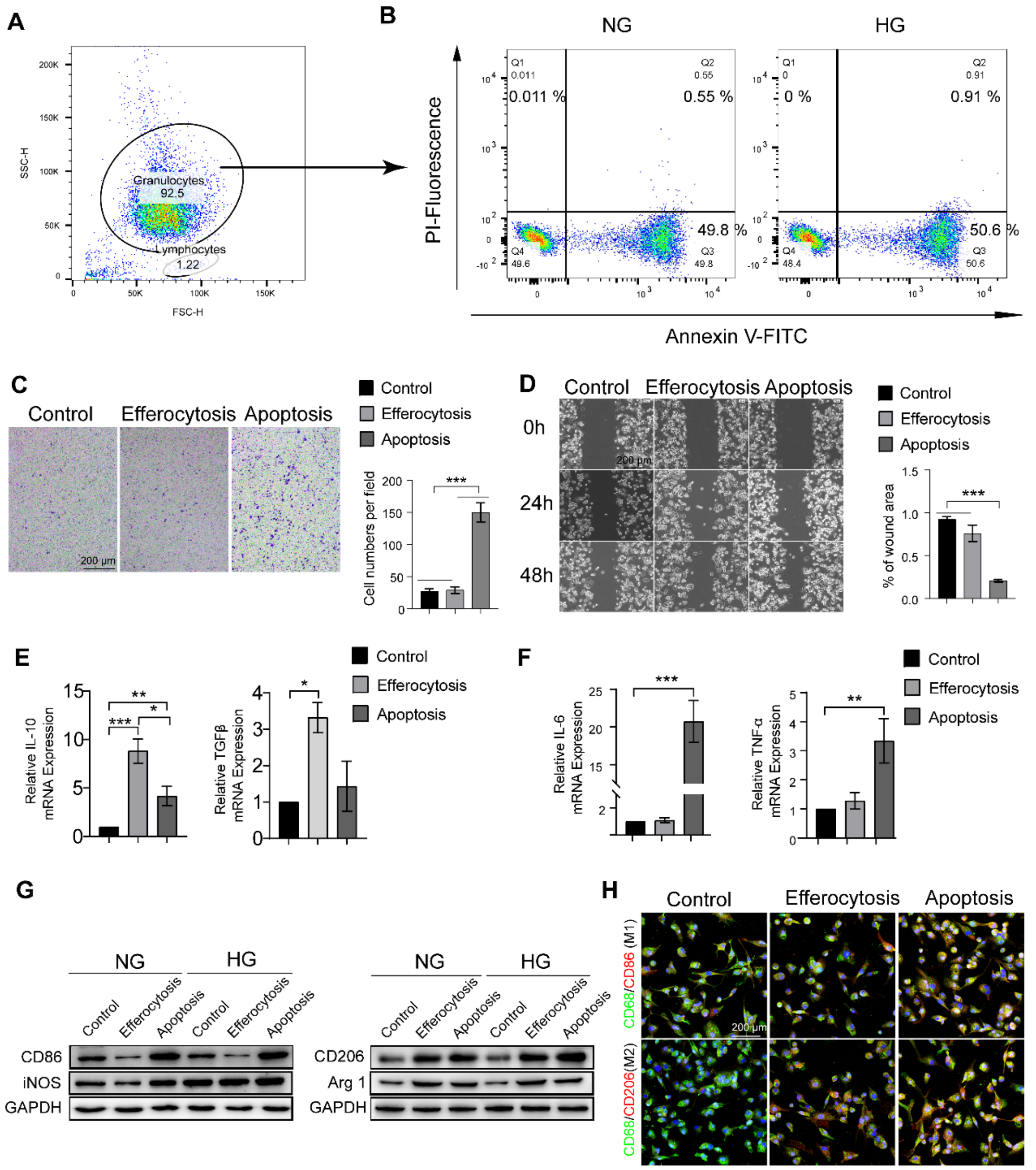

Fig. 3. Promoting efferocytosis of apoptotic neutrophils inhibits abnormal infiltration and phenotypic polarization of macrophages.

(A) Neutrophils were isolated by erythrocyte sedimentation and centrifugation. (B) Flow cytometry showed that the spontaneous apoptosis of neutrophils was not increased after 6 hours of high glucose culture in vitro. (C) Transwell migration in macrophages after efferocytosis, and apoptotic stimulation. (D) Wound healing assays were used to investigate the migratory ability of macrophages stimulated by efferocytosis, and apoptosis. (E) Efferocytosis promotes macrophages to secrete anti-inflammatory mediators such as IL-10 and TGF-β. (F) Macrophages co-cultured with

apoptotic neutrophils secrete a large number of inflammatory mediators, such as IL-6 and TNF-α. (G-H) WB and immunofluorescence analysis of macrophage differentiation stimulated by efferocytosis, and apoptosis. The results were presented as means ± S.D. *$p < 0.05$; **$p < 0.01$; ***$p > 0.001$ by ANOVA or 2-tailed, unpaired Student's t test.

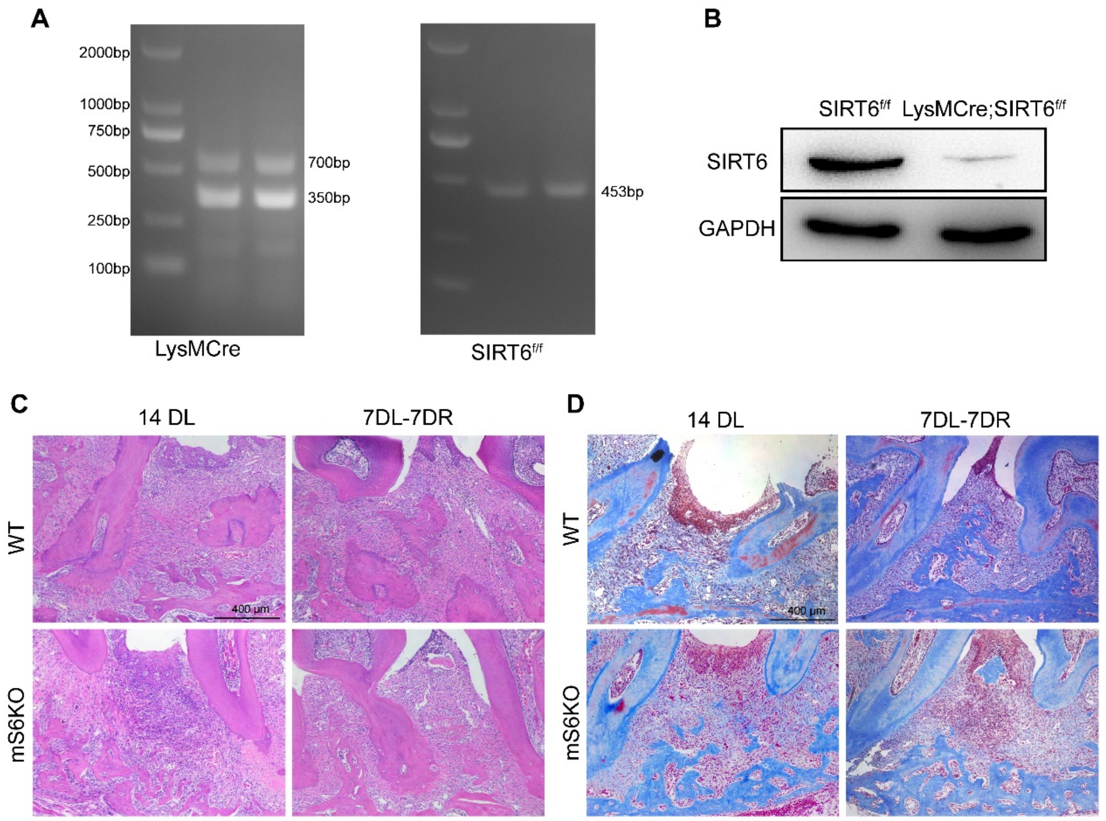

Fig. 4. Myeloid-specific SIRT6 knockout promotes alveolar bone loss in ligature-induced periodontitis.

(A) Representative PCR image of tail genotyping of each group. (B) The expression of SIRT6 in BMMs from mS6KO mice was detected by Western blot analysis. (C) Representative images of H&E-stained paraffin sections. (D) Representative images of Masson-stained paraffin sections.

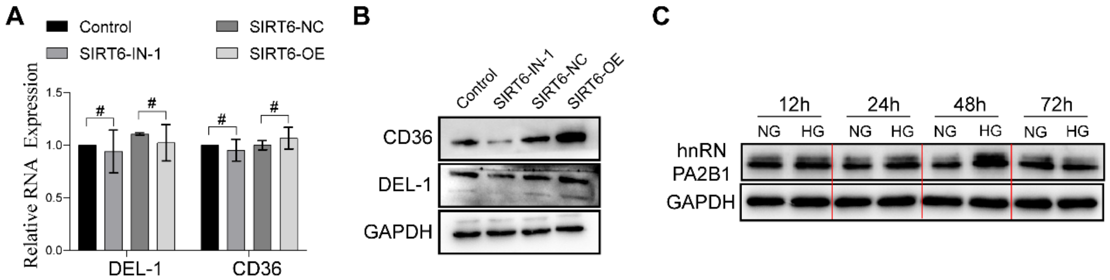

Fig. 5. High glucose and SIRT6 overexpression increased the expression of hnRNPA2B1, DEL-1, and CD36.

(A) The expression of hnRNPA2B1 was detected by WB in macrophages after high glucose stimulation. (B) The RT-qPCR results showed that there was no significant change in the RNA level of DEL-1 and CD36 after SIRT6 inhibition and overexpression. (C) WB results showed that the expression of DEL-1 and CD36 was low after SIRT6 inhibition, and the expression of DEL-1 and CD36 was high after SIRT6 overexpression.

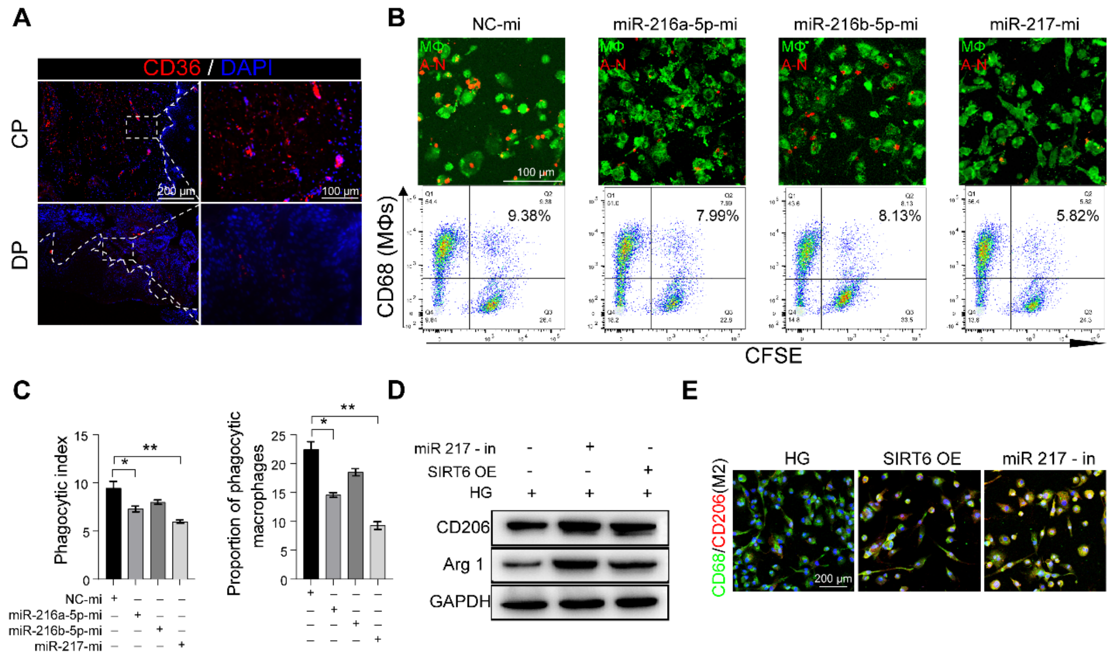

Fig. 6. miR-216/217 cluster negatively regulates macrophage efferocytosis.

(A) The expression of CD36 in diabetic periodontitis was decreased compared with periodontitis. (B-C) Phagocytic index of macrophages following exposure to the miR-217-5p and miR-216a-5p and miR-216b-5p mimic. (D-E) Under high glucose conditions, WB and immunofluorescence analysis of macrophage differentiation after restoring macrophage efferocytosis by over-expression SIRT6 or knockdown miR-217. The results were presented as means ± S.D. *p < 0.05; **p < 0.01 by ANOVA or 2-tailed, unpaired Student's t test. The white dotted line indicates the boundary between the root and the alveolar bone and the gingiva.

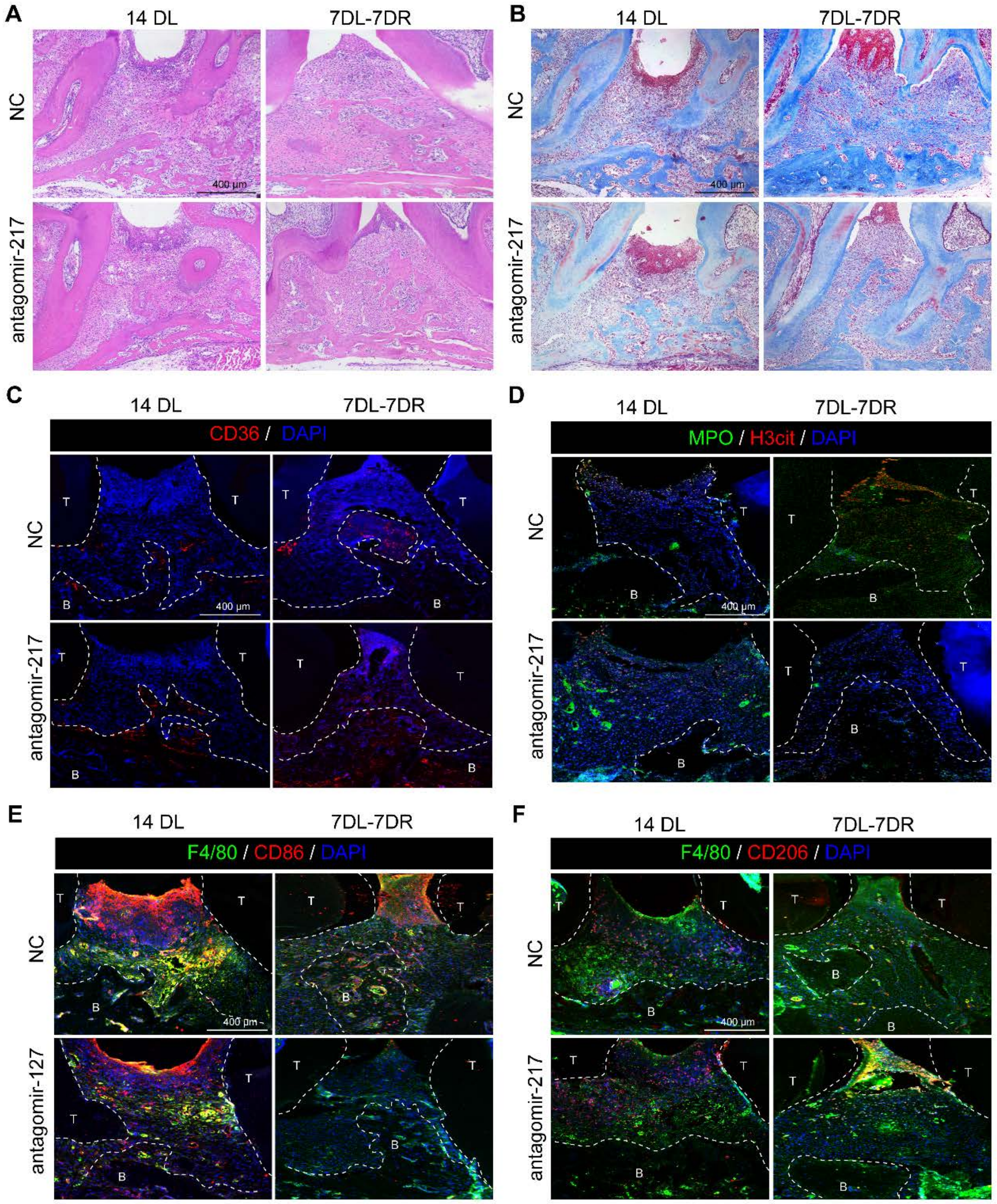

Fig. 7. Antagomir-217 injection promotes the resolution of ligature-induced diabetic periodontitis and reduces alveolar bone loss.

(A) Representative images of H&E-stained paraffin sections in the periodontium of NC and antagomir-217 treatment mice. (B) Representative images of Masson-stained paraffin sections in the periodontium of NC and antagomir-217 treatment mice. (C) Representative images of the expression of CD36 in the periodontium of NC and antagomir-217 treatment mice. (D) Representative images of H3cit (red) and MPO (green) positive NETs in the periodontium of NC and antagomir-217 treatment mice.

(E-F) Representative images of M1 and M2 in the periodontium of NC and antagomir-217 treatment mice. The white dotted line indicates the boundary between the root and the alveolar bone and the gingiva.

Supplementary materials and methods

miRNA microarray

Total RNA was extracted from macrophages and macrophages after SIRT6 inhibition and met the RNA quality control requirements of the array. The Personalbio Company (Personalbio, Nanjing) performed the miRNA microarray assay. Hiseq single end mode is adopted for sequencing and genome alignment analysis using miRDeep2 (Mackowiak SD, 2011) software. Analyze the data obtained by Affymetrix Expression Console software according to the MAS5 method. The spots with a | log2 ratio| ≥ 0.263 and a $p < 0.05$ were selected for analysis.

Wound Healing and Cellular Transwell assay

Cell migration was determined by scratch wound healing and Cellular transwell assay. Macrophages were inoculated into six-well plates, subjected to serum starvation in a serum-free medium for 24 h, and then an artificial wound was created using a 200 mL pipette tip. Images were taken at 0 h, 24 h, and 48 h using an inverted microscope. For in vitro invasion assay, approximately 1×10^5 cells of differentiated macrophages were plated in serum-free media in the top chamber (Millipore, USA). Medium supplemented with 10% FBS was added to the lower chamber. The cells were then incubated at 37 °C in 5% CO_2 for 48 h. The chambers were then fixed with 4% paraformaldehyde, and stained with crystal violet, and the number of macrophages was counted under a microscope.

RNA extraction and quantitative RT-PCR (qRT-PCR)

Total RNA was extracted using TRIzol according to the guideline and reversely transcribed into complementary DNA (cDNA) by a HiScript II Q RT SuperMix for qPCR kit (Vazyme, China). The RT of miRNA was conducted using the tailing method. For Real-time qPCR, the StepOne Plus RT PCR System (Thermo, USA) was used by SYBR Green (Vazyme, China). The relative expression normalized to GAPDH or U6 was analyzed with the formula $2^{-\Delta\Delta Ct}$.

Western blot

Western blot was performed using methods described previously. RIPA buffer (Beyotime, Shanghai, China) containing 10 mM protease inhibitor (PMSF; Beyotime)

was used to lyse cells. Protein lysate was separated by 10–15% SDS-PAGE gels and transferred proteins to polyvinylidene fluoride (PVDF) membranes (Millipore). Membranes were blocked by 5% fat-free milk for 2 h and incubated with primary antibodies overnight. After washing with TBST three times, the membranes were then incubated with the appropriate HRP-conjugated secondary antibody (1:10,000). Protein bands were detected with an ECL detection kit. ImageJ software was performed to analyze the Western blot.

High glucose stimulation of macrophages

In this experiment, 1640 medium with three Glucose concentrations was used: normal glucose concentration (NG): 5.5mmol/L, ordinary 1640 medium: 11.11 mmol/L, and high glucose 1640 medium (HG): 25mmol/L. NG and HG are prepared from glucose-free 1640 medium RPMI culture medium (Gibco, Waltham, MA, USA) and glucose, respectively. After the glucose is dissolved, filter sterilization. Conventional culture of THP-1 and induction of macrophages were performed using an ordinary 1640 medium, and subsequent stimulation with different sugar concentrations was performed using a 1640 medium with corresponding sugar concentrations.

Transfection of plasmids, microRNA mimics/inhibitors, and small interfering RNAs (siRNAs)

has miR-217b, has miR-216a-5p, has miR-216b-5p mimic/inhibitor, siRNAs targeting hnRNPA2B1, and SIRT6 overexpression plasmid was designed and synthesized by RiboBio (Guangzhou, China). siRNA, plasmid, and miR-217b, miR-216a-5p, and miR-216b-5p mimic/inhibitor were transfected into cells using Lipofectamine 2000 (Invitrogen, Carlsbad, CA, USA). SIRT6 was inhibited using the SIRT6 inhibitor OSS_128167 (SIRT6-IN-1) at 100 μM/mL (Selleck, USA). All sequences are listed (Table S2).

Analysis of single-cell RNA-seq and GO analysis.

Single-cell RNA-seq raw data generated in skin specimens from DFU-Non-healers (DFU, diabetic foot ulceration) and foot skin of healthy non-diabetic subjects were retrieved from NCBI Gene Expression Omnibus (GEO). DFU-Non-healers labeling GSM5050530, GSM5050533, GSM5050557, GSM5050558, and

GSM5050563, foot skin of healthy non-diabetic labeling GSM5050534, GSM5050538, GSM5050534, GSM5050534, GSM5050552, GSM5050552, GSM5050556, GSM5050556, GSM5050556, GSM5050568, and GSM5050574. Download the Sequence Read Archive (SRA) raw data from the NCBI server. Single-cell RNA-seq analysis and GO analysis based on previous studies.

antagomir-217

antagomir-217 and antagomir-NC were designed and synthesized by RiboBio (Guangzhou, China). A microsyringe (5μL Hamilton, Switzerland) was used for injection. From the first day of ligation, the injection was once every three days, with a total of 4 injections. The corresponding drugs were injected into the mesial and distal gingiva of the mouse's buccal and palatal sides of the maxillary second molar using a micro-syringe, respectively. The injection dose of antagomir-217 and antagomir-NC was 10 nmol per mouse.

Supplementary Material 1

Abbreviations

Teeth were numbered according to the International Federation of Dentists (FDI). BI: bleeding index, PD: probing depth.

Chronic periodontitis:

Patient 1

Age: 42 Gender: male Diagnosis: Chronic periodontitis;

22: PD: 4mm, BI: 2; **27**: PD: 7mm, BI: 3; **45**, PD: 4mm; BI: 2; **46**: PD: 8mm, BI: 3.

22、27、45、46 alveolar bone loss >70% of the root.

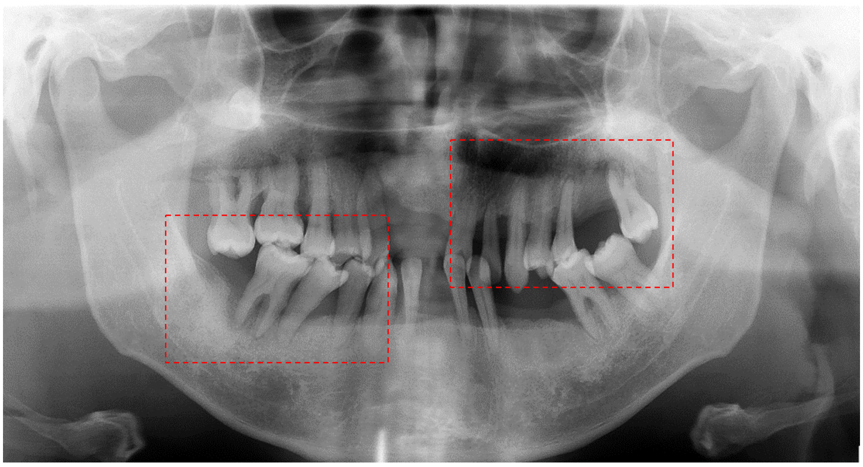

Patient 2

Age: 65 Gender: male Diagnosis: Chronic periodontitis;

41: PD: 4mm, BI: 2; **42**: PD: 4mm, BI: 2; **46**: PD: 8mm; BI: 3; **47**: PD: 8mm; BI: 3; **48**: PD: 4mm, BI: 1.

41、42、47、46、48 alveolar bone loss >80% of the root.

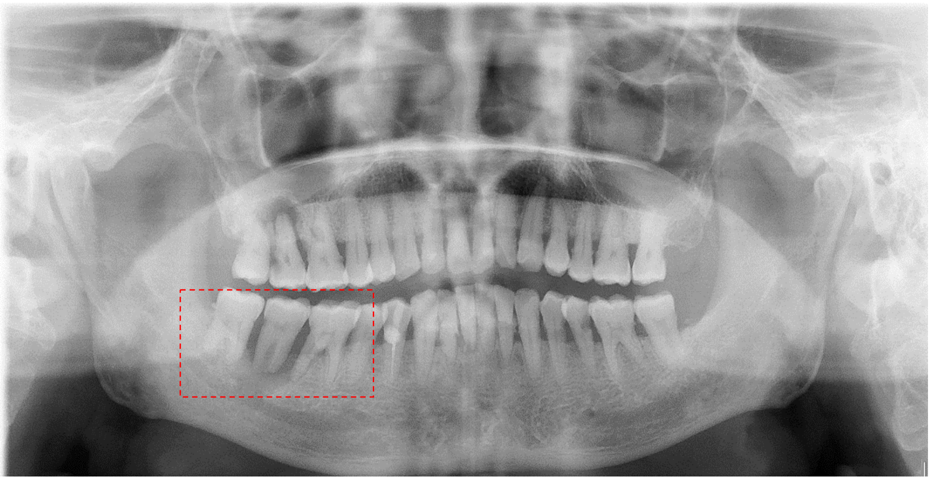

Patient 3

Age: 42 Gender: male Diagnosis: Chronic periodontitis;

36: PD: 8mm; BI: 3; **37**: PD: 6mm, BI: 3.

36、37 alveolar bone loss >70% of the root.

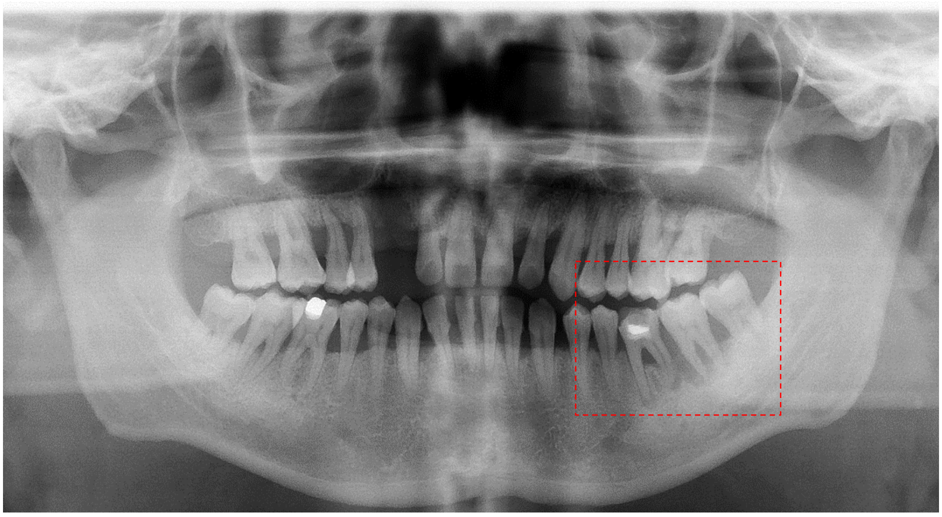

Patient 4

Age: 38 Gender: male Diagnosis: Chronic periodontitis;

34: PD: 5mm; BI: 3; **35**: PD: 4mm, BI: 2; **36**: PD: 8mm, BI: 3; **37**: PD: 5mm, BI: 2.

34、35、36、37 alveolar bone loss >80% of the root.

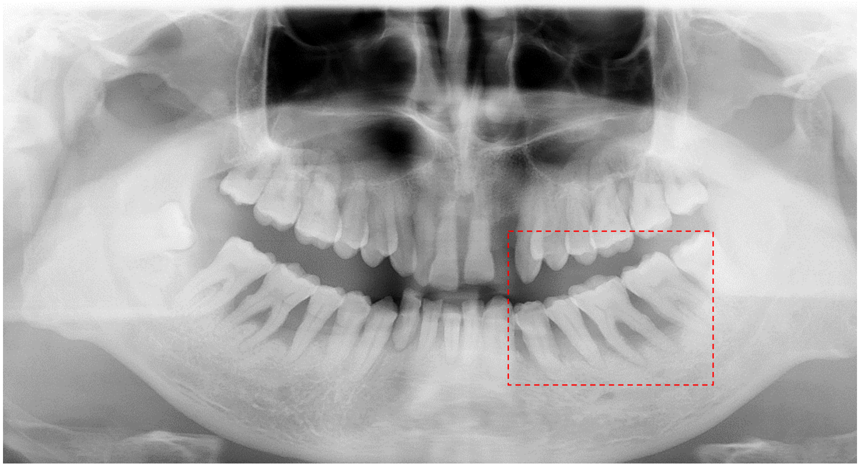

Patient 5

Age: 32 Gender: male Diagnosis: 1. Chronic periodontitis; 2. Cyst of Maxilla

12, PD: 4mm; BI: 2; **13**: PD: 5mm, BI: 3; **14**: PD: 4mm, BI: 2.

12、13、14 alveolar bone loss >80% of the root.

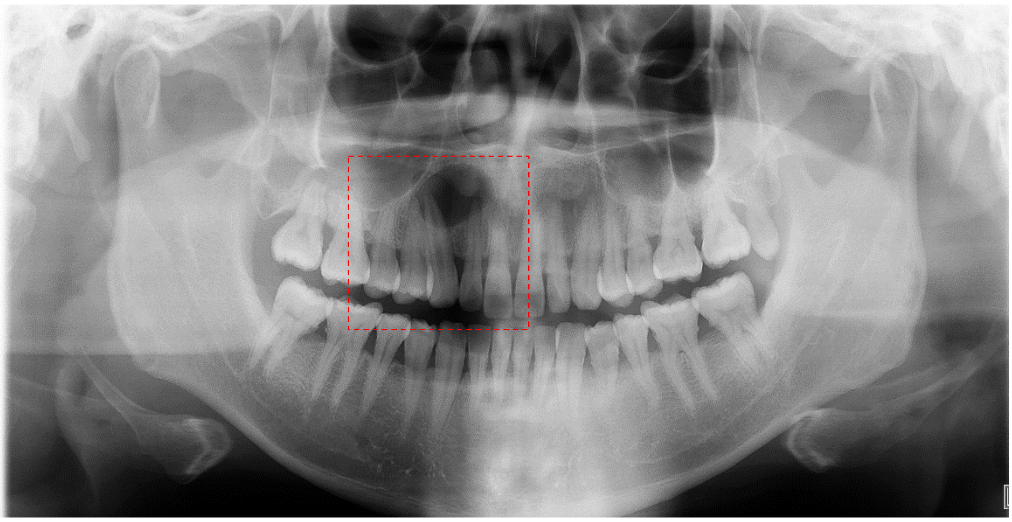

Diabetes periodontitis:

Patient 1

Age: 42 Gender: female Diagnosis: 1. Chronic periodontitis; 27, Periodontal abscess;
25: PD: 6mm, BI: 2; **27**: PD: 8mm, BI: 3; **28**, PD: 7mm; BI: 2.
25, 27, and 28 alveolar bone loss >80% of the root.

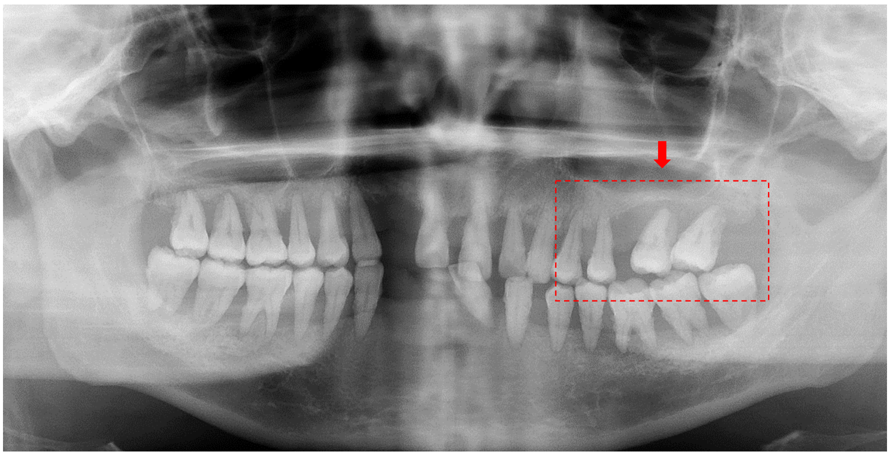

Patient 2

Age: 59 Gender: female Diagnosis: 1. Chronic periodontitis;
35: PD: 6mm, BI: 2; **36**: PD: 7mm, BI: 3; **37**: PD: 8mm, BI: 3; **38**, PD: 5mm; BI: 2.

35, 36, 37, and 38 alveolar bone loss >80% of the root.

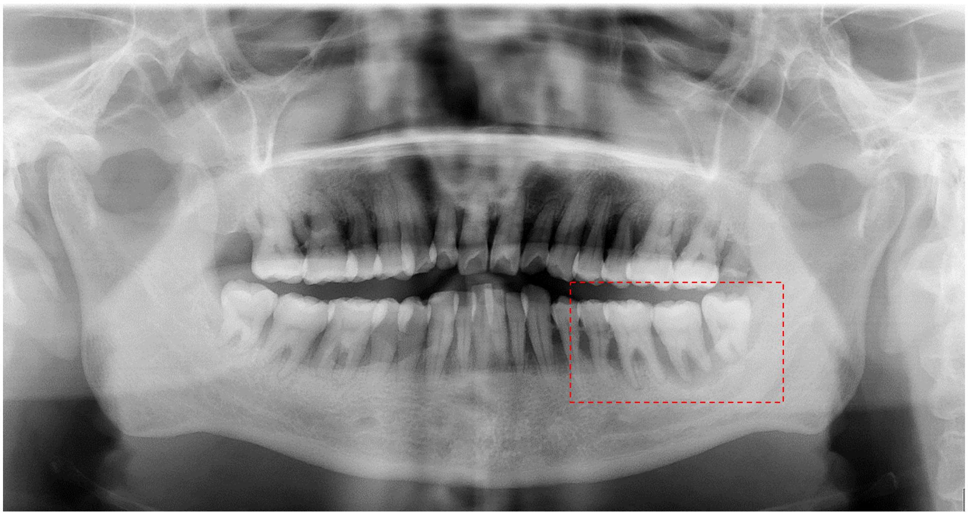

Patient 3

Age: 55 Gender: male Diagnosis: 1. Chronic periodontitis; 31, 41 Immediate Implant;
31: PD: 4mm, BI: 2; **41**: PD: 5mm, BI: 3.
31 and 41 alveolar bone loss >80% of the root.

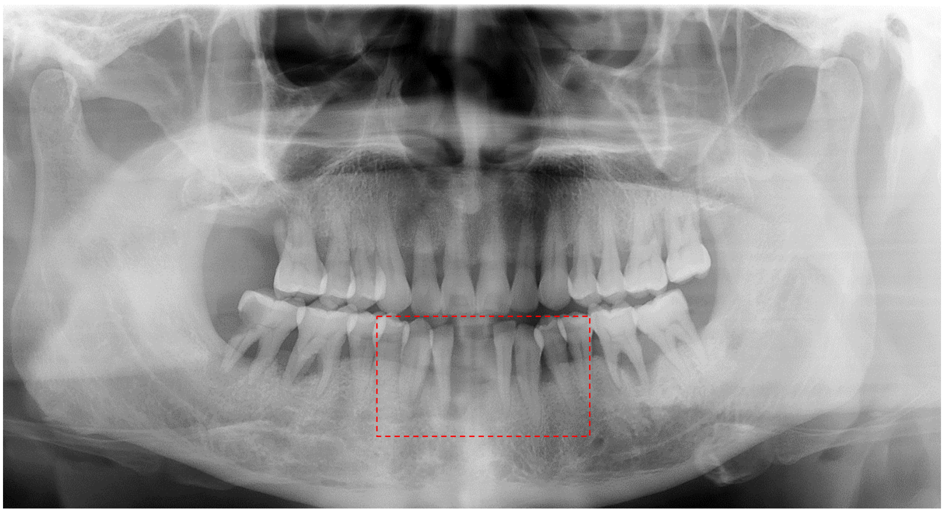

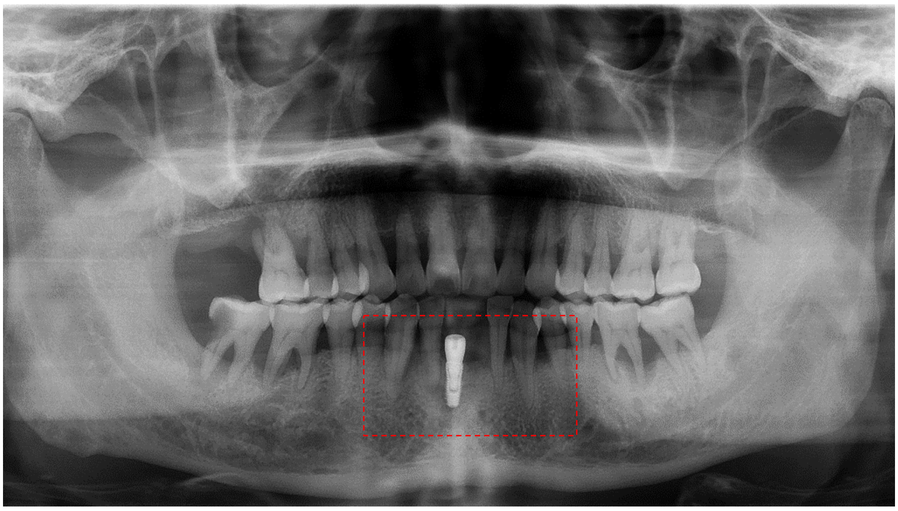

Patient 4

Age: 28   Gender: male   Diagnosis: 1. Chronic periodontitis; 2. Cyst of Maxilla

11: PD: 5mm, BI: 2; **12**: PD: 6mm, BI: 3; **13**: PD: 4mm, BI: 3; **21**, PD: 5mm; BI: 2.

11, 12, 13, and 21 alveolar bone loss >70% of the root.

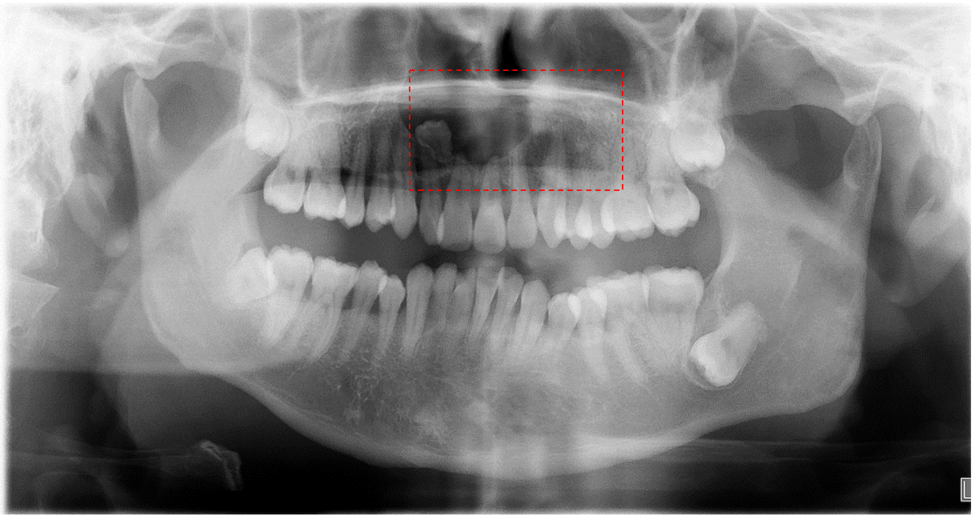

Patient 5

Age: 65   Gender: male   Diagnosis: 1. Chronic periodontitis; 27. Mandibular cyst;

42: PD: 4mm, BI: 2; **43**: PD: 5mm, BI: 3; **44**, PD: 4mm; BI: 2; **45**, PD: 5mm; BI: 2; **46**, PD: 4mm; BI: 1;

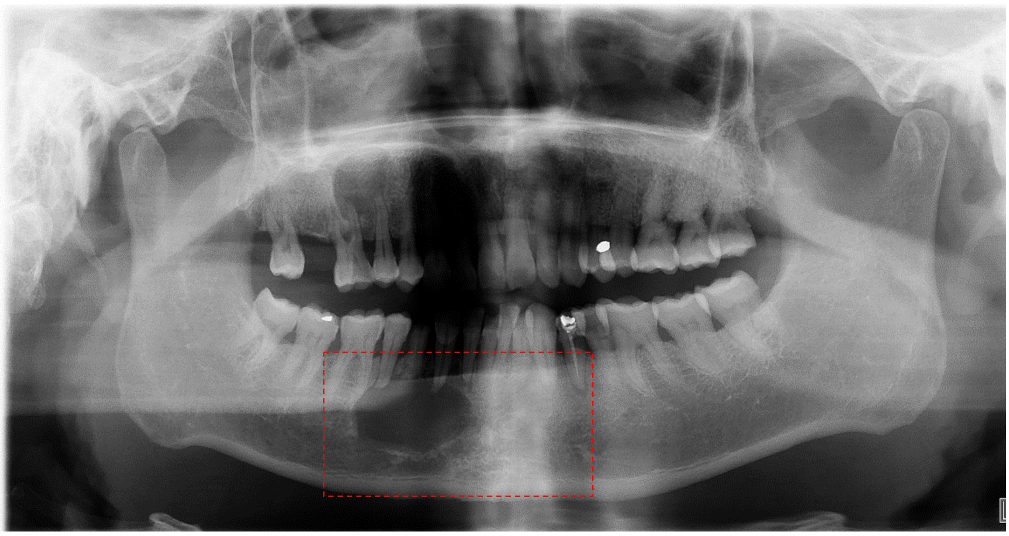

Supplementary Table Legends

Table S1. Antibody list.

Table S2. List of all prime-, probe-, and plasmid-related sequences.
